# Geometrical variations of two manganese(II) complexes with closely related quinoline-based tripodal ligands

**DOI:** 10.1107/S2056989021009786

**Published:** 2021-09-28

**Authors:** Steven T. Frey, Jasper G. Ballot, Allison Hands, Haley A. Cirka, Katheryn C. Rinaolo, Nich N. Phalkun, Manpreet Kaur, Jerry P. Jasinski

**Affiliations:** a Department of Chemistry, Skidmore College, 815 North Broadway, Saratoga Springs, NY 12866, USA; b Department of Chemistry, Keene State College, 229 Main Street, Keene, NH, 03435-2001, USA

**Keywords:** crystal structure, manganese(II), tripodal ligand, quinoline, 6-coordinate, *cis/trans*

## Abstract

Structural analyses of the compounds di-μ-acetato-κ^4^ *O*:*O*′-bis­{[2-meth­oxy-*N*,*N*-bis­(quinolin-2-ylmeth­yl)ethanamine-κ^4^ *N*,*N*′,*N*′′,*O*]manganese(II)} bis­(tetra­phen­yl­borate) di­chloro­methane 1.45-solvate, [Mn_2_(C_23_O_2_)_2_(C_23_H_23_N_3_O)_2_](C_24_H_20_B)·1.45CH_2_Cl_2_ or [Mn(DQMEA)(μ-OAc)_2_Mn(DQMEA)](BPh_4_)_2_·1.45CH_2_Cl_2_ or **[1]**(BPh_4_)_2_·1.45CH_2_Cl_2_, and (acetato-κ*O*)[2-hy­droxy-*N*,*N*-bis(quinolin-2-ylmeth­yl)ethanamine-κ^4^ *N*,*N*′,*N*′′,*O*](methanol-κ*O*)manganese(II) tetra­phenyl­borate methanol monosolvate, [Mn(CH_3_COO)(C_22_H_21_N_3_O)(CH_3_OH)](C_24_H_20_B)·CH_3_OH or [Mn(DQEA)(OAc)(CH_3_OH)]BPh_4_·CH_3_OH or **[2]**BPh_4_·CH_3_OH, by single-crystal X-ray diffraction reveal distinct differences in the geometry of coordination of the tripodal DQEA and DQMEA ligands to Mn^II^ ions. In the asymmetric unit, compound **[1]**(BPh_4_)_2_·(CH_2_Cl_2_)_1.45_ crystallizes as a dimer in which each manganese(II) center is coordinated by the central amine nitro­gen, the nitro­gen atom of each quinoline group, and the meth­oxy-oxygen of the tetra­dentate DQMEA ligand, and two bridging-acetate oxygen atoms. The symmetric Mn^II^ centers have a distorted, octa­hedral geometry in which the quinoline nitro­gen atoms are *trans* to each other resulting in co-planarity of the quinoline rings. For each Mn^II^ center, a coordinated acetate oxygen participates in C—H⋯O hydrogen-bonding inter­actions with the two quinolyl moieties, further stabilizing the *trans* structure. Within the crystal, weak *π*–*π* stacking inter­actions and inter­molecular cation–anion inter­actions stabilize the crystal packing. In the asymmetric unit, compound **[2]**BPh_4_·CH_3_OH crystallizes as a monomer in which the manganese(II) ion is coordinated to the central nitro­gen, the nitro­gen atom of each quinoline group, and the alcohol oxygen of the tetra­dentate DQEA ligand, an oxygen atom of OAc, and the oxygen atom of a methanol ligand. The geometry of the Mn^II^ center in **[2]**BPh_4_·CH_3_OH is also a distorted octa­hedron, but the quinoline nitro­gen atoms are *cis* to each other in this structure. Hydrogen bonding between the acetate oxygen atoms and hydroxyl (O—H⋯O) and quinolyl (C—H⋯O and N—H⋯O) moieties of the DQEA ligand stabilize the complex in this *cis* configuration. Within the crystal, dimerization of complexes occurs by the formation of a pair of inter­molecular O3—H3⋯O2 hydrogen bonds between the coordinated hydroxyl oxygen of the DQEA ligand of one complex and an acetate oxygen of another. Additional hydrogen-bonding and inter­molecular cation–anion inter­actions contribute to the crystal packing.

## Abstract 2

The crystal structures of two manganese(II) complexes have been determined. The manganese(II) centers of each structure are six-coordinate with a distorted octa­hedral geometry. Although the bis­(quinolin-2-ylmeth­yl)ethanamine ligands differ only by a methyl group, the structure of one complex is dimeric with bridging acetate ligands and exhibits a *trans* coordination and coplanarity of the quinolyl moieties, while the second complex is monomeric with a *cis* coordination of the quinolyl groups.

## Chemical context   

Synthetic manganese(II) compounds have gained attention in recent years owing to their anti­oxidant (Signorella *et al.*, 2018 ▸; Batinić-Haberle *et al.*, 2010 ▸, 2014 ▸; Iranzo, 2011 ▸; Bani & Bencini, 2012 ▸; Miriyala *et al.*, 2012 ▸; Policar, 2016 ▸), anti­cancer (Icsel *et al.*, 2020 ▸; Prihantono *et al.*, 2020 ▸; Liu *et al.*, 2015 ▸; Wang *et al.*, 2014 ▸; Zhou *et al.*, 2011 ▸), anti­bacterial (Saha *et al.*, 2020 ▸; Maurya *et al.*, 2011 ▸, Dong *et al.*, 2017 ▸), optoelectronic (Qin *et al.*, 2020 ▸), catalytic (Sarma *et al.*, 2019 ▸), and MRI enhancement (Wang *et al.*, 2018 ▸, Boros *et al.*, 2015 ▸, Gale *et al.*, 2015 ▸) properties. Manganese(II) tends to be less toxic than other metal ions (Iranzo, 2011 ▸; Bani & Bencini, 2012 ▸), can often reversibly access the Mn^III^ oxidation state, and exhibits luminescence in some instances (Qin *et al.*, 2020 ▸). The ability to form stable, efficacious Mn^II^ compounds for these applications is dependent upon the nature of the ligands employed, their coord­in­ating atoms, and other groups that can alter the geometry, bulkiness, and/or optical properties of the compound (Signorella *et al.*, 2018 ▸, Policar, 2016 ▸, Qin *et al.*, 2020 ▸).
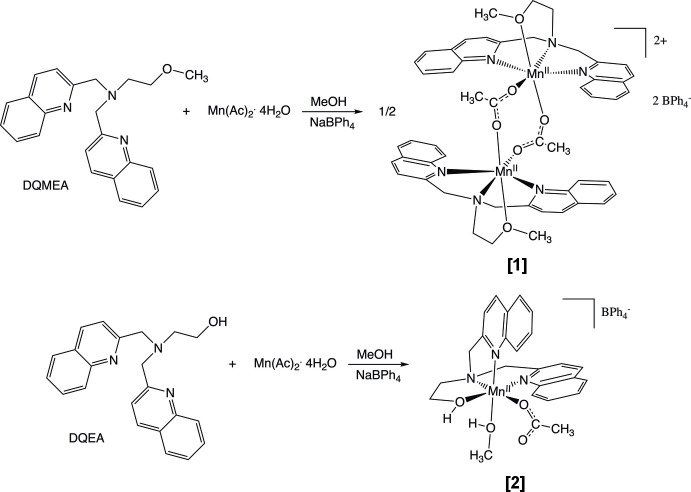


We have recently begun to study Mn^II^ compounds with tetra­dentate, tripodal ligands (Frey, Li *et al.*, 2018 ▸; Frey, Ramirez *et al.*, 2018 ▸). These ligands are readily synthesized to provide a variety of N and O donors and other groups that can potentially alter the structural and/or electronic properties of the Mn^II^ center. Quinoline groups, for example, provide bulkiness that can lead to distorted coordination geometries, potentially altering the coordination number, redox potential, substrate specificity, and/or photophysical properties of a complex. Quinoline ring systems are also the basis for a number of biologically active mol­ecules, suggesting that their presence might lead to medicinally-relevant compounds (Kakoulidou *et al.*, 2021 ▸). We report here the synthesis and structural characterization of [Mn(DQMEA)(μ-OAc)_2_Mn(DQMEA)](BPh_4_)_2_·(CH_2_Cl_2_)_1.45_, **[1]**(BPh_4_)_2_·1.45CH_2_Cl_2_ where DQMEA = 2-meth­oxy-*N*,*N*-bis­(quinolin-2-ylmeth­yl)ethanamine, OAc = acetate, BPh_4_ = tetra­phenyl­borate and [Mn(DQEA)(OAc)(CH_3_OH)]BPh_4_·CH_3_OH, **[2]**BPh_4_·CH_3_OH where DQEA = 2-hy­droxy-*N*,*N*-bis­(quinolin-2-yl­meth­yl)ethanamine). These compounds are prepared in a two-step reaction (see reaction scheme) in which mangan­ese(II) acetate is reacted with either DQMEA or DQEA in methanol, followed by anion exchange with sodium tetra­phenyl­borate. The resulting complexes demonstrate how minor alterations in ligand structure can result in significant differences in the complex structure.

## Structural commentary   

**Figure 1:**
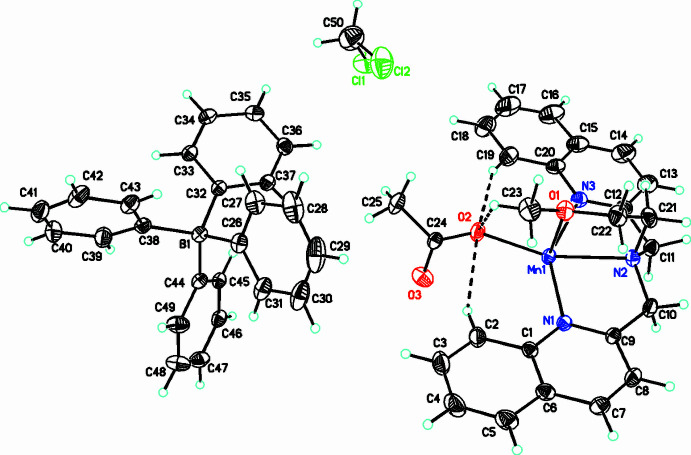
The title compound **[1]**(BPh_4_)_2_·(CH_2_Cl_2_)_1.45_ with displacement ellipsoids drawn at the 30% probability level. Only the major disorder components for the di­chloro­methane solvent are shown. Dashed lines indicate intra­molecular weak C—H⋯O inter­actions influencing the stability of the complex conformation.

**Figure 2:**
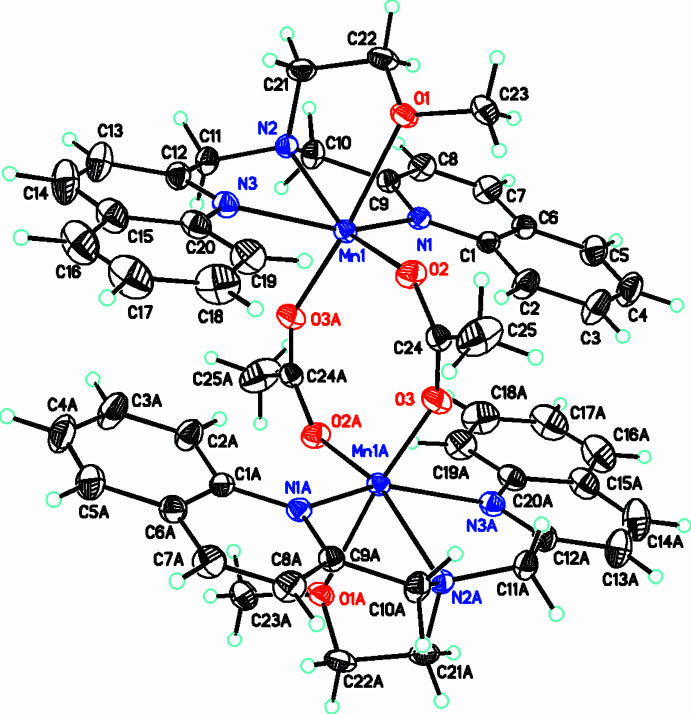
Structure of the [Mn(DQMEA)(μ-OAc)_2_Mn(DQMEA)]^2+^ complex [DQMEA = 2-meth­oxy-*N*,*N*-bis­(quinolin-2-ylmeth­yl)ethanamine, OAc = acetate] with atom labels. Displacement ellipsoids drawn at the 30% probability level.

Compound **[1]**(BPh_4_)_2_·(CH_2_Cl_2_)_1.45_ crystallizes in the triclinic space group *P* 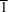 (Fig. 1 ▸). The structure reveals a dimeric [Mn(DQMEA)(μ-OAc)_2_Mn(DQMEA)]^2+^ cation, **[1]** (Fig. 2 ▸) balanced by the presence of tetra­phenyl borate anions. The manganese(II) ions are hexa­coordinate with a distorted octa­hedral geometry. While this is a standard coordination number for transition metal cations, manganese(II) complexes with N-donor ligands are often hepta­coordinate (Frey, Li *et al.*, 2018 ▸; Deroche *et al.*, 1996 ▸; Policar *et al.*, 2001 ▸; Lessa *et al.*, 2007 ▸; Dees *et al.*, 2007 ▸; Wu *et al.*, 2010 ▸; Lieb *et al.*, 2013 ▸). The presence of the bulky quinoline rings in this compound may restrict the coordination number to six in **[1]**. The DQMEA ligands are tetra­dentate, with the central N2 and two quinolyl nitro­gen atoms (N1 and N3) in the same octa­hedral plane and the meth­oxy oxygen (O1) located perpendicular to this nitro­gen plane. This configuration of the DQMEA ligand results in the quinoline groups binding Mn^II^ *trans* to each other, and in coplanarity of their rings. Hydrogen-bonding inter­actions between quinolyl hydrogens and an acetate oxygen, C—H⋯O, further stabilize this *trans* configuration (Table 3 ▸). Oxygens from two bridging acetate ions make up the final two coordinating atoms, with O2 *trans* to the central N2 nitro­gen of DQMEA and O3 *trans* to the meth­oxy oxygen, O1. Distortion of the octa­hedral geometry of the coordination sphere is caused by the bite angles of the DQMEA ligand. For example, the five-membered metallacycles formed by coord­ination of quinoline nitro­gens and central nitro­gen of DQMEA, produce bond angles, N2—Mn1—N3 and N2—Mn1—N1, of 73.25 (5) and 75.56 (5)°, respectively, which are significantly reduced from 90° (Table 1 ▸). This results in a *trans* N1—Mn1—N3 angle of 148.35 (5)°. Likewise, the bond angle formed by *cis* coordination of the meth­oxy oxygen of DQMEA and central nitro­gen, N2—Mn1—O1 is 75.32 (5)°. The remaining *trans* bond angles, O2—Mn1—N2 and O3^1^—Mn1—O1 are 157.89 (5) and 163.58 (5)°, respectively. The Mn—O and Mn—N bond lengths for the neutral DQMEA ligand fall in the range 2.27–2.36 Å, which is typical of manganese(II) complexes (Deroche *et al.*, 1996 ▸; Policar *et al.*, 2001 ▸; Lessa *et al.*, 2007 ▸; Dees *et al.*, 2007 ▸; Wu *et al.*, 2010 ▸; Lieb *et al.*, 2013 ▸). However, the Mn1—O2 and Mn1—O3^1^ acetate bond lengths, 2.0617 (13) and 2.0908 (14) Å, are significantly shorter.

**Figure 3:**
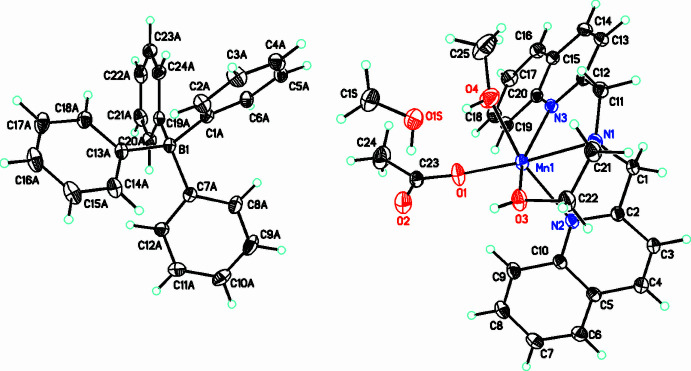
The title compound **[2]**(BPh_4_)·CH_3_OH with displacement ellipsoids drawn at the 30% probability level. (Only the major disorder components for the hy­droxy­ethyl fragment are shown.)

The compound **[2]**BPh_4_·CH_3_OH crystallizes in the monoclinic space group *P*2_1_/c. The structure of this compound consists of the [Mn(DQEA)(OAc)(CH_3_OH)]^+^ monocation, **[2]**, tetra­phenyl borate counter-ion, and a methanol solvent mol­ecule (Fig. 3 ▸). The Mn^II^ ion is hexa­coordinate with a distorted octa­hedral geometry. As with **[1]**, the bulky quinoline groups likely prevent a seven-coordinate species from forming. The DQEA ligand is tetra­dentate, but the quinolyl nitro­gen atoms in this structure, N2 and N3, are *cis* to each other, and the rings are therefore not co-planar. The central nitro­gen of DQEA, N1 and the quinolyl nitro­gens occupy an octa­hedral face, while the alcohol oxygen, O3 is *trans* to the quinolyl nitro­gen N3. In addition to the DQEA ligand, a monodentate acetate oxygen, O1 is *trans* to the central nitro­gen of DQEA, while a methanol oxygen, O4 occupies a position *trans* to the quinolyl nitro­gen, N2. Like DQMEA in **[1]**, binding constraints of the DQEA ligand in **[2]** result in significant distortions of the octa­hedral geometry of the coordination sphere. Bond angles involving the central nitro­gen of DQEA and quinolyl nitro­gens, N1—Mn1—N2 and N1—Mn1—N3 are 75.63 (5) and 73.81 (5)°, respectively (Table 2 ▸). The alcohol oxygen and quinolyl nitro­gen that are *trans* to each other, form a bond angle with manganese, O3—Mn1—N3 of 149.83 (12)°. The remaining *trans* bond angles, O1—Mn1—N1 and N2—Mn1—O4 are 175.54 (6) and 161.38 (6)°, respectively.

The *cis* coordination of DQEA to Mn(II) in **[2]** may result from a hydrogen-bonding network involving the alcohol and quinolyl groups of DQEA and the acetate ligand, O—H⋯O and C—H⋯O (Table 4 ▸). A *trans* configuration of DQEA, like that of DQMEA in **[1]** would swing the alcohol hydrogen up and away from the acetate ligand, preventing this hydrogen-bonding inter­action. Additional O—H⋯O hydrogen bonds in **[2]**BPh_4_·CH_3_OH, between methanol mol­ecules themselves and with the acetate ligand, provide further stabilization of the structure. This *cis* structure observed in **[2]**BPh_4_·CH_3_OH may not be favorable with the DQMEA ligand, since the meth­oxy methyl group would disrupt this hydrogen-bonding network.

## Supra­molecular features   

**Figure 4:**
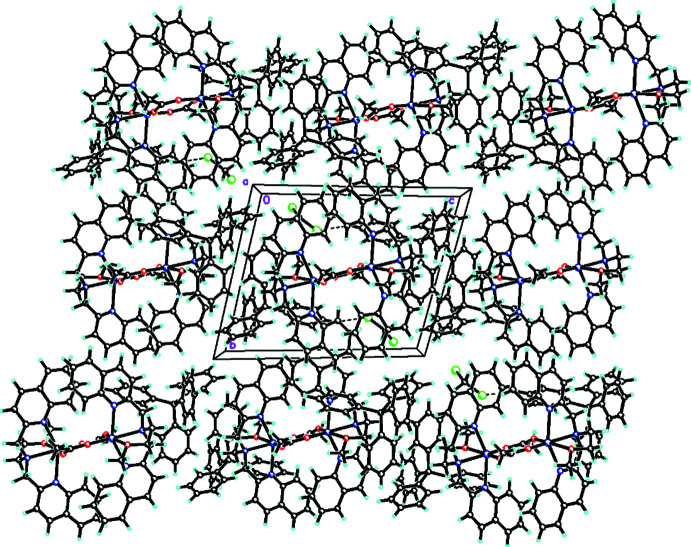
A view along the *a* axis of the crystal packing of **[1]**(BPh_4_)_2_·(CH_2_Cl_2_)_1.45_ with dashed lines indicating weak C—H⋯Cl inter­actions. Minor disordered solvate mol­ecules were omitted for clarity.

Within the crystal of **[1]**(BPh_4_)_2_·(CH_2_Cl_2_)_1.45_, no classical inter­molecular hydrogen bonding inter­actions were found. The crystal packing (Fig. 4 ▸) is primarily stabilized by weak C29—H29⋯Cl2 inter­actions (Table 3 ▸) and *π*–*π* stacking inter­actions between nearby benzene rings (*Cg*7⋯*Cg*6) of a quinoline group (where *Cg*7 and *Cg*6 are the centroids of the C15–C120 and C1–C6 rings, respectively). In addition, a network of weak C—H⋯*π* (C8—H8⋯*Cg*11, *X*—H, *π* = 78°; C11—H11*B*⋯*Cg*11, *X*—H, *π* = 59°, C23—H23*B*⋯*Cg*9, *X*—H, *π* = 72°, where *Cg*9 and *Cg*11 are the centroids of the C32–C37 and C44–C49 rings, respectively) inter­molecular cation–anion inter­actions (Table 3 ▸) are also present and contribute additionally to the crystal packing.

**Figure 5:**
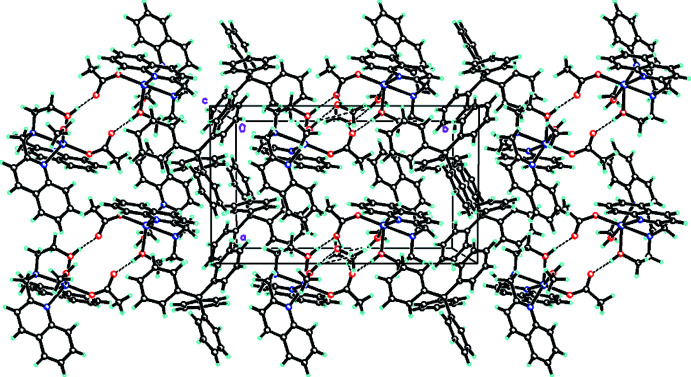
A view along the *c* axis of the crystal packing of **[2]**BPh_4_·CH_3_OH. The intra­molecular and inter­molecular O—H⋯O and C—H⋯O hydrogen bonds (Table 4 ▸) are shown as dashed lines. Solvate mol­ecules were omitted for clarity.

Within the crystal of **[2]**BPh_4_·CH_3_OH, dimerization of complexes occurs by the formation of a pair of inter­molecular O3—H3⋯O2 hydrogen bonds (Table 4 ▸) between the coordinated hydroxyl oxygen of DQEA ligand of one complex and an acetate oxygen of another (Fig. 5 ▸), forming an 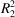
(12) ring-motif inter­action. In addition, the methanol solvent mol­ecule forms strong O—H⋯O hydrogen bonds (Table 4 ▸) with the coordinated methanol and acetate ligands of the cationic complex, forming an 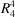
(16) ring motif influencing the crystal packing. Weak C11—H11*A*⋯*Cg*12 (*X*—H, *π* = 58°; where *Cg*12 is the centroid of the C13*A*–C18*A* ring) inter­molecular cation–anion inter­actions (Table 4 ▸) are also present and contribute additionally to the crystal packing.

## Database survey   

To the best of our knowledge, structures of the manganese(II) compounds described herein have not been reported previously. We have previously reported the structure of a mononuclear copper(II) complex with DQMEA (Frey, Ramirez *et al.*, 2018 ▸). In this structure, the DQMEA ligand is tetra­dentate with a *tris* configuration of the quinoline groups as observed in **[1]**. A search of the Cambridge Crystallographic Database (updated in May 2021; Groom *et al.*, 2016 ▸) revealed a related manganese(II) complex with a penta­dentate, tripodal ligand containing two methyl quinolyl groups and an imine thiol­ate group (Coggins & Kovacs, 2011 ▸). This ligand binds the Mn^II^ ion in a trigonal–bipyramidal geometry with the quinoline rings *cis* to each other in the equatorial plane, similar to **[2]**.

## Synthesis and crystallization   

All chemicals were obtained from commercial sources and used without further purification. The water used was deion­ized. The ^1^H NMR spectra were recorded with a JEOL JNM-ECZ400s NMR spectrometer and referenced against chloro­form. IR spectra were recorded with a Perkin Elmer Spectrum 100 FT–IR.

**2-Meth­oxy-*****N***,***N*****-bis­(quinolin-2-ylmeth­yl)ethanamine (DQMEA)**. In a 250 ml round-bottom flask, 5 g (23 mmol) of 2-chlormethyl­quinoline hydro­chloride was dissolved in 10 ml of H_2_O and cooled to 273 K in an ice bath. A solution of 1.9 g (47 mmol) of NaOH in 10 ml of H_2_O was added dropwise with stirring. Following this, a solution of 0.9 g (12 mmol) of 2-meth­oxy­ethyl­amine in 10 ml of CH_2_Cl_2_ was added. The reaction mixture was then removed from the ice bath, and brought to reflux for 7 days. The mixture was then cooled to room temperature, and the CH_2_Cl_2_ layer was separated, washed twice with brine, and dried over anhydrous sodium sulfate. The solution was then filtered, and the filtrate was chromatographed on alumina (chromatographic grade, 80–200 mesh) eluting with 20:1 CH_2_Cl_2_/methanol. Fractions were collected that produced a single spot by TLC on alumina plates (eluting with 100:1, CH_2_Cl_2_/methanol) with an *R*_F_ value of 0.33. Rotary evaporation of these fractions gave 2.4 g (58%) of a light-yellow solid. ^1^H NMR (CDCl_3_, 400 MHz) δ 2.87 (*t*, 2H), 3.30 (*s*, 3H), 3.54 (*t*, 2H), 4.06 (*s*, 4H), 7.48 (*t*, 2H), 7.65 (*t*, 2H), 7.75 (*m*, 4H), 8.01 (*d*, 2H), 8.10 (*d*, 2H).

**2-Hy­droxy-*****N***,***N*****-bis­(quinolin-2-ylmeth­yl)ethanamine (DQEA)**. In a 100 ml round-bottom flask, 2.5 g (12 mmol) of 2-chlormethyl­quinoline hydro­chloride was dissolved in 10 ml of H_2_O and cooled to 273 K in an ice bath. A solution of 0.95 g (24 mmol) of NaOH in 10 ml of H_2_O was added dropwise with stirring. Following this, a solution of 0.36 g (6.0 mmol) of ethano­lamine in 10 ml of CH_2_Cl_2_ was added. The reaction mixture was then removed from the ice bath, and brought to reflux for 7 days. The mixture was then cooled to room temperature, and the CH_2_Cl_2_ layer was separated, washed twice with brine, and dried over anhydrous sodium sulfate. The solution was then filtered, and the filtrate was chromatographed on alumina (chromatographic grade, 80–200 mesh) eluting with 100:1 CH_2_Cl_2_/methanol. Fractions were collected that produced a single spot by TLC on alumina plates (eluting with 100:1, CH_2_Cl_2_/methanol) with an *R*_F_ value of 0.33. Rotary evaporation of these fractions gave 0.70 g (20%) of a light-yellow solid. ^1^H NMR (CDCl_3_, 400 MHz) δ 3.02 (*t*, 2H), 3.54 (*t*, 2H), 4.17 (*s*, 4H), 7.51 (*m*, 4H), 7.74 (*m*, 4H), 8.07 (*m*, 4H).

**[Mn(DQMEA)(μ-OAc)_2_Mn(DQMEA)](BPh_4_)_2_**. In a 100 ml round-bottom flask, 0.20 g (0.56 mmol) of DQEA was dissolved in 10 ml of methanol. To this solution, 0.14 g (0.58 mmol) of manganese(II) acetate tetra­hydrate was added, and the solution was brought to reflux for 30 minutes. A solution of 0.19 g (0.56 mmol) of sodium tetra­phenyl­borate in 10 ml of methanol was then added dropwise to the warm reaction mixture. The solution was then cooled in a refrigerator to promote crystallization of the compound. After several hours, the reaction mixture was filtered to produce light-yellow microcrystals that were washed twice with cold methanol and air dried to give 0.36 g (82%) of product. Recrystallization of 20 mg of this product in a mixture of di­chloro­methane and methanol gave crystals suitable for X-ray diffraction. These crystals had an IR spectrum identical to the original product. IR (ATR, cm^−1^) 2800–3200 (aromatic C—H, *w*), 1600 (C—O, *s*), 1425 (C—O, *s*), 731 (BPh_4_, *s*), 704 (BPh_4_, *s*).

**[Mn(DQEA)(OAc)(CH_3_OH)]BPh_4_·CH_3_OH**. In a 100 ml round-bottom flask, 0.20 g (0.58 mmol) of DQEA was dissolved in 10 ml of methanol. To this solution, 0.14 g (0.58 mmol) of manganese(II) acetate tetra­hydrate was added, and the solution was brought to reflux for 30 minutes. A solution of 0.20 g (0.58 mmol) of sodium tetra­phenyl­borate in 10 ml of methanol was then added dropwise to the warm reaction mixture. The solution was then cooled in a refrigerator to promote crystallization of the compound. After several hours, the reaction mixture was filtered to produce light yellow microcrystals that were washed twice with cold methanol and air dried to give 0.31 g (69%) of product. Recrystallization of 20 mg of this product in a mixture of di­chloro­methane and methanol gave crystals suitable for X-ray diffraction. These crystals had an IR spectrum identical to the original product. IR (ATR, cm^−1^) 2800–3200 (aromatic C—H, *w*), 1578 (C—O, *s*), 1427 (C—O, *s*), 736 (BPh_4_, *s*), 700 (BPh_4_, *s*).

## Refinement   

Crystal data, data collection and structure refinement details for **[1]**(BPh_4_)_2_·(CH_2_Cl_2_)_1.45_ and **[2]**BPh_4_·CH_3_OH are summarized in Table 5 ▸. For **[1]**(BPh_4_)_2_·(CH_2_Cl_2_)_1.45_, all H atoms were positioned geometrically and refined using a riding model: C—H = 0.93–0.99 Å, with *U*_iso_(H) = 1.2*U*_eq_(C) or 1.5*U*_eq_(C-meth­yl). Idealized methyl groups were refined as rotating groups. A solvate methyl­ene chloride mol­ecule was refined as threefold disordered. All C—Cl bond distances were restrained to be the same within a standard deviation of 0.02 Å. *U* ^ij^ components of ADPs were restrained to be similar to each other (SIMU command, esd = 0.01 Å^2^). Occupancies were not constrained to unity and refined to 0.401 (3), 0.234 (4) and 0.090 (4). In **[2]**BPh_4_·CH_3_OH, the ethanol group of C21, C22 and O3 was found to be disordered. Bond distances and angles of major and minor moiety were restrained to be similar to each other (SAME and SADI commands, esd = 0.02 Å). *U* ^ij^ components of ADPs were restrained to be similar to each other (SIMU command, esd = 0.01 Å^2^). The hy­droxy H atoms (O3—H3, O3*B*—H3*B*, O4—H4) were located in a difference-Fourier map and refined with the distance restraint O—H = 0.8 (2) Å and with *U*_iso_(H) = 1.5*U*_eq_(O). C-bound H atoms were positioned geometrically and refined as riding: C—H = 0.95–0.99 Å with *U*_iso_(H) = 1.2*U*_eq_(C) or 1.5*U*_eq_(C-meth­yl). Idealized methyl groups were refined as rotating groups. An idealized tetra­hedral OH group was also refined as a rotating group: O1*S*(H1*S*).

## Supplementary Material

Crystal structure: contains datablock(s) 1, 2. DOI: 10.1107/S2056989021009786/zl5024sup1.cif

Structure factors: contains datablock(s) 1. DOI: 10.1107/S2056989021009786/zl50241sup2.hkl

Structure factors: contains datablock(s) 2. DOI: 10.1107/S2056989021009786/zl50242sup3.hkl

CCDC references: 2110882, 2110881

Additional supporting information:  crystallographic information; 3D view; checkCIF report

## Figures and Tables

**Table 1:** Selected geometric parameters (Å, °) for **[1]**(BPh_4_)_2_·1.45CH_2_Cl_2_ [Chem scheme1]

Mn1—O1	2.3225 (12)	Mn1—N1	2.3179 (14)
Mn1—O2	2.0617 (13)	Mn1—N2	2.2730 (14)
Mn1—O3^i^	2.0908 (14)	Mn1—N3	2.3588 (16)
			
N2—Mn1—N3	73.25 (5)	N2—Mn1—O1	75.32 (5)
N2—Mn1—N1	75.56 (5)	O2—Mn1—N2	157.89 (6)
N1—Mn1—N3	148.35 (5)	O3^i^—Mn1—O1	163.58 (6)

Symmetry code: (i) -x, -y+1, -z+1.

**Table 2:** Selected geometric parameters (Å, °) for **[2]**BPh_4_·CH_3_OH[Chem scheme1]

Mn1—O1	2.0551 (14)	Mn1—N1	2.2787 (15)
Mn1—O3	2.182 (7)	Mn1—N2	2.3167 (15)
Mn1—O3*B*	2.13 (3)	Mn1—N3	2.2664 (14)
Mn1—O4	2.3190 (16)		
			
N1—Mn1—N2	75.63 (5)	O1—Mn1—N1	175.54 (6)
N1—Mn1—N3	73.81 (5)	N2—Mn1—O4	161.38 (6)
O3—Mn1—N3	149.83 (12)		

**Table 3:** Hydrogen-bond geometry (Å, °) for **[1]**(BPh_4_)_2_·1.45CH_2_Cl_2_ [Chem scheme1] *Cg*9 and *Cg*12 are the centroids of the C32–C37 and C44–C49 rings, respectively.

*D*—H⋯*A*	*D*—H	H⋯*A*	*D*⋯*A*	*D*—H⋯*A*
C2—H2⋯O2	0.95	2.49	3.366 (3)	154
C19—H19⋯O2	0.95	2.31	3.199 (3)	155
C23—H23*A*⋯O2	0.98	2.31	3.1767 (2)	119
C29—H29⋯Cl2^ii^	0.95	2.65	3.5305 (2)	155
C8—H8⋯*Cg*11^iii^	0.95	2.68	3.5556 (2)	153
C11—H11*B*⋯*Cg*11^iv^	0.99	2.81	3.7195 (2)	152
C23—H23*B*⋯*Cg*9	0.98	2.78	3.7034 (2)	157

Symmetry codes: (ii) -x+1, -y+1, -z+1; (iii) -x+1, -y+1, -z; (iv) x+1, y, z.

**Table 4:** Hydrogen-bond geometry (Å, °) for **[2]**BPh_4_·CH_3_OH[Chem scheme1]

*D*—H⋯*A*	*D*—H	H⋯*A*	*D*⋯*A*	*D*—H⋯*A*
O3—H3⋯O2^i^	0.85 (2)	1.79 (2)	2.631 (8)	170 (4)
O3*B*—H3*B*⋯O2^i^	0.84 (2)	1.87 (8)	2.65 (3)	152 (14)
O4—H4⋯O1*S*	0.89 (2)	1.77 (2)	2.646 (2)	168 (3)
C9—H9⋯O1	0.95	2.43	3.325 (3)	157
C17—H17⋯O1*S* ^ii^	0.95	2.73	3.364 (3)	125
C18—H18⋯O1*S* ^ii^	0.95	2.73	3.367 (2)	125
C19—H19⋯O1	0.95	2.39	3.183 (2)	141
C25—H25*A*⋯N3	0.98	2.79	3.387 (3)	120
O1*S*—H1*S*⋯O2^i^	0.84	1.92	2.691 (2)	151

Symmetry codes: (i) -x, -y+1, -z+1; (ii) x+1, y, z.

**Table 5:** Experimental details

	**[1]**(BPh_4_)_2_·1.45CH_2_Cl_2_	**[2]**BPh_4_·CH_3_OH
Crystal data		
Chemical formula	[Mn_2_(C_2_H_3_O_2_)_2_(C_23_H_23_N_3_O)_2_](C_24_H_20_B)·1.45CH_2_Cl_2_	[Mn(C_2_H_3_O_2_)(C_22_H_21_N_3_O)(CH_4_O)](C_24_H_20_B)·CH_4_O
*M*_r_	1704.59	840.69
Crystal system, space group	Triclinic, *P*\overline{1}	Monoclinic, *P*2_1_/*c*
Temperature (K)	173	173
*a*, *b*, *c* (Å)	11.6553 (5), 13.6846 (7), 16.1109 (6)	10.3504 (3), 17.4824 (5), 23.9618 (9)
α, β, γ (°)	96.842 (4), 105.959 (3), 111.907 (4)	90, 96.222 (3), 90
*V* (Å^3^)	2220.29 (18)	4310.3 (3)
*Z*	1	4
Radiation type	Mo *K*α	Mo *K*α
μ (mm^−1^)	0.43	0.36
Crystal size (mm)	0.32 × 0.26 × 0.18	0.34 × 0.28 × 0.26

Data collection		
Diffractometer	Rigaku Oxford Diffraction Gemini Eos	Rigaku Oxford Diffraction Gemini Eos
Absorption correction	Multi-scan (*CrysAlis PRO*; Rigaku OD, 2019 ▸)	Multi-scan (*CrysAlis PRO*; Rigaku OD, 2019 ▸)
*T*_min_, *T*_max_	0.819, 1.000	0.845, 1.000
No. of measured, independent and observed [*I* > 2σ(*I*)] reflections	27481, 14664, 10475	31473, 14443, 10348
*R*_int_	0.027	0.032
(sin θ/λ)_max_ (Å^−1^)	0.762	0.765

Refinement		
*R*[*F*^2^ > 2σ(*F* ^2^)], *wR*(*F* ^2^), *S*	0.054, 0.145, 1.02	0.054, 0.149, 1.04
No. of reflections	14664	14443
No. of parameters	600	582
No. of restraints	141	85
H-atom treatment	H-atom parameters constrained	H atoms treated by a mixture of independent and constrained refinement
Δρ_max_, Δρ_min_ (e Å^−3^)	0.98, −0.38	0.67, −0.44

Computer programs: *CrysAlis PRO* (Rigaku OD, 2019 ▸), *SHELXT* (Sheldrick, 2015 ▸ ▸a), *SHELXL* (Sheldrick, 2015 ▸ ▸b), and *OLEX2* (Dolomanov *et al.*, 2009 ▸).

## supplementary crystallographic information

### Di-µ-acetato-κ4O:O'-bis{[2-methoxy-N,N-bis(quinolin-2-ylmethyl)ethanamine-κ4N,N',N'',O]manganese(II)} bis(tetraphenylborate) dichloromethane 1.45-solvate (1) Crystal data

**Table:**

[Mn_2_(C_2_H_3_O_2_)_2_(C_23_H_23_N_3_O)_2_](C_24_H_20_B)·1.45CH_2_Cl_2_	*Z* = 1
*M**_r_* = 1704.59	*F*(000) = 891
Triclinic, *P*1	*D*_x_ = 1.275 Mg m^−^^3^
*a* = 11.6553 (5) Å	Mo *K*α radiation, λ = 0.71073 Å
*b* = 13.6846 (7) Å	Cell parameters from 6992 reflections
*c* = 16.1109 (6) Å	θ = 3.1–31.9°
α = 96.842 (4)°	µ = 0.43 mm^−^^1^
β = 105.959 (3)°	*T* = 173 K
γ = 111.907 (4)°	Prism, clear colourless
*V* = 2220.29 (18) Å^3^	0.32 × 0.26 × 0.18 mm

### Di-µ-acetato-κ4O:O'-bis{[2-methoxy-N,N-bis(quinolin-2-ylmethyl)ethanamine-κ4N,N',N'',O]manganese(II)} bis(tetraphenylborate) dichloromethane 1.45-solvate (1) Data collection

**Table:**

Rigaku Oxford Diffraction Gemini Eos diffractometer	14664 independent reflections
Radiation source: fine-focus sealed X-ray tube	10475 reflections with *I* > 2σ(*I*)
Detector resolution: 16.0416 pixels mm^-1^	*R*_int_ = 0.027
ω scans	θ_max_ = 32.8°, θ_min_ = 2.6°
Absorption correction: multi-scan (CrysAlisPro; Rigaku OD, 2019)	*h* = −17→14
*T*_min_ = 0.819, *T*_max_ = 1.000	*k* = −20→19
27481 measured reflections	*l* = −23→23

### Di-µ-acetato-κ4O:O'-bis{[2-methoxy-N,N-bis(quinolin-2-ylmethyl)ethanamine-κ4N,N',N'',O]manganese(II)} bis(tetraphenylborate) dichloromethane 1.45-solvate (1) Refinement

**Table:**

Refinement on *F*^2^	Primary atom site location: dual
Least-squares matrix: full	Hydrogen site location: inferred from neighbouring sites
*R*[*F*^2^ > 2σ(*F*^2^)] = 0.054	H-atom parameters constrained
*wR*(*F*^2^) = 0.145	*w* = 1/[σ^2^(*F*_o_^2^) + (0.0585*P*)^2^ + 0.9487*P*] where *P* = (*F*_o_^2^ + 2*F*_c_^2^)/3
*S* = 1.02	(Δ/σ)_max_ = 0.001
14664 reflections	Δρ_max_ = 0.98 e Å^−^^3^
600 parameters	Δρ_min_ = −0.38 e Å^−^^3^
141 restraints	

### Di-µ-acetato-κ4O:O'-bis{[2-methoxy-N,N-bis(quinolin-2-ylmethyl)ethanamine-κ4N,N',N'',O]manganese(II)} bis(tetraphenylborate) dichloromethane 1.45-solvate (1) Special details

**Table:**

Geometry. All esds (except the esd in the dihedral angle between two l.s. planes) are estimated using the full covariance matrix. The cell esds are taken into account individually in the estimation of esds in distances, angles and torsion angles; correlations between esds in cell parameters are only used when they are defined by crystal symmetry. An approximate (isotropic) treatment of cell esds is used for estimating esds involving l.s. planes.

### Di-µ-acetato-κ4O:O'-bis{[2-methoxy-N,N-bis(quinolin-2-ylmethyl)ethanamine-κ4N,N',N'',O]manganese(II)} bis(tetraphenylborate) dichloromethane 1.45-solvate (1) Fractional atomic coordinates and isotropic or equivalent isotropic displacement parameters (Å^2^)

**Table:**

	*x*	*y*	*z*	*U*_iso_*/*U*_eq_	Occ. (<1)
C50	0.2765 (8)	0.1499 (8)	0.2170 (7)	0.087 (2)	0.401 (3)
H50A	0.310430	0.093680	0.222114	0.104*	0.401 (3)
H50B	0.314159	0.193635	0.178427	0.104*	0.401 (3)
Cl1	0.1035 (4)	0.0871 (4)	0.1687 (4)	0.0912 (13)	0.401 (3)
Cl2	0.3236 (2)	0.2312 (2)	0.31916 (15)	0.0994 (10)	0.401 (3)
C50B	0.1821 (13)	0.0765 (17)	0.2361 (13)	0.110 (3)	0.234 (4)
H50C	0.161913	0.089629	0.291088	0.132*	0.234 (4)
H50D	0.149835	−0.003348	0.217034	0.132*	0.234 (4)
Cl1B	0.0714 (8)	0.1029 (7)	0.1592 (7)	0.106 (3)	0.234 (4)
Cl2B	0.3482 (8)	0.1217 (5)	0.2742 (4)	0.121 (2)	0.234 (4)
C50C	0.120 (3)	0.044 (4)	0.210 (2)	0.098 (4)	0.091 (4)
H50E	0.111892	−0.031315	0.202229	0.117*	0.091 (4)
H50F	0.067467	0.052816	0.246824	0.117*	0.091 (4)
Cl1C	0.0580 (17)	0.0658 (14)	0.1071 (15)	0.121 (4)	0.091 (4)
Cl2C	0.2853 (19)	0.1342 (16)	0.2661 (13)	0.113 (3)	0.091 (4)
Mn1	0.09233 (2)	0.45986 (2)	0.62585 (2)	0.02693 (7)	
O1	0.29639 (12)	0.48106 (12)	0.71832 (8)	0.0371 (3)	
O2	0.18228 (14)	0.48215 (12)	0.53151 (8)	0.0394 (3)	
O3	0.09615 (13)	0.54513 (13)	0.42244 (10)	0.0483 (4)	
N1	0.15837 (14)	0.62702 (11)	0.72088 (9)	0.0278 (3)	
N2	0.05875 (14)	0.41899 (12)	0.75235 (9)	0.0292 (3)	
N3	−0.01211 (15)	0.26857 (12)	0.59563 (10)	0.0328 (3)	
C1	0.22911 (16)	0.72862 (14)	0.71239 (11)	0.0288 (3)	
C2	0.2634 (2)	0.73924 (17)	0.63578 (13)	0.0437 (5)	
H2	0.235771	0.676298	0.589900	0.052*	
C3	0.3360 (3)	0.8393 (2)	0.62682 (17)	0.0581 (6)	
H3	0.359397	0.845201	0.574992	0.070*	
C4	0.3766 (3)	0.93357 (19)	0.69306 (18)	0.0594 (6)	
H4	0.427293	1.002669	0.685928	0.071*	
C5	0.3439 (2)	0.92634 (17)	0.76695 (16)	0.0498 (5)	
H5	0.371590	0.990495	0.811584	0.060*	
C6	0.26878 (19)	0.82404 (15)	0.77838 (12)	0.0353 (4)	
C7	0.2307 (2)	0.81229 (17)	0.85354 (13)	0.0437 (5)	
H7	0.253913	0.874609	0.898758	0.052*	
C8	0.1608 (2)	0.71178 (17)	0.86105 (12)	0.0410 (4)	
H8	0.134139	0.703068	0.911408	0.049*	
C9	0.12753 (17)	0.61981 (14)	0.79355 (11)	0.0292 (3)	
C10	0.04934 (19)	0.50950 (15)	0.80483 (12)	0.0360 (4)	
H10A	0.080307	0.509994	0.868655	0.043*	
H10B	−0.044479	0.496451	0.787555	0.043*	
C11	−0.06460 (18)	0.32018 (15)	0.72553 (12)	0.0348 (4)	
H11A	−0.140053	0.339350	0.706239	0.042*	
H11B	−0.069498	0.288954	0.777493	0.042*	
C12	−0.07526 (18)	0.23631 (15)	0.65099 (13)	0.0356 (4)	
C13	−0.1571 (2)	0.12665 (18)	0.64192 (18)	0.0543 (6)	
H13	−0.196943	0.106657	0.685119	0.065*	
C14	−0.1784 (3)	0.0501 (2)	0.5708 (2)	0.0642 (7)	
H14	−0.234905	−0.023909	0.563105	0.077*	
C15	−0.1173 (2)	0.08009 (18)	0.50898 (16)	0.0498 (5)	
C16	−0.1365 (3)	0.0053 (2)	0.43211 (19)	0.0667 (8)	
H16	−0.194530	−0.069156	0.420691	0.080*	
C17	−0.0738 (3)	0.0384 (2)	0.37522 (18)	0.0708 (8)	
H17	−0.089249	−0.012700	0.323412	0.085*	
C18	0.0142 (3)	0.1473 (2)	0.39118 (16)	0.0658 (7)	
H18	0.058621	0.169348	0.350618	0.079*	
C19	0.0365 (2)	0.2227 (2)	0.46546 (14)	0.0505 (5)	
H19	0.097415	0.296177	0.476515	0.061*	
C20	−0.03004 (19)	0.19127 (16)	0.52453 (13)	0.0383 (4)	
C21	0.16945 (19)	0.39647 (18)	0.80239 (13)	0.0405 (4)	
H21A	0.157389	0.323842	0.772881	0.049*	
H21B	0.168339	0.395084	0.863466	0.049*	
C22	0.30084 (19)	0.48029 (19)	0.80770 (12)	0.0425 (5)	
H22A	0.317164	0.552820	0.840554	0.051*	
H22B	0.372491	0.461419	0.839134	0.051*	
C23	0.41726 (19)	0.5603 (2)	0.71646 (16)	0.0505 (5)	
H23A	0.412101	0.558094	0.654474	0.076*	
H23B	0.490552	0.544134	0.747434	0.076*	
H23C	0.431810	0.632837	0.746210	0.076*	
C24	0.17232 (17)	0.50770 (15)	0.45824 (11)	0.0306 (3)	
C25	0.2625 (3)	0.4928 (3)	0.41239 (19)	0.0715 (8)	
H25A	0.322654	0.468549	0.450229	0.107*	
H25B	0.313689	0.562083	0.401453	0.107*	
H25C	0.210272	0.438180	0.355498	0.107*	
C26	0.45814 (17)	0.73445 (16)	0.27058 (12)	0.0356 (4)	
C27	0.5447 (2)	0.6969 (2)	0.32062 (14)	0.0482 (5)	
H27	0.576336	0.654627	0.290197	0.058*	
C28	0.5860 (2)	0.7196 (2)	0.41342 (17)	0.0672 (8)	
H28	0.644722	0.692757	0.444992	0.081*	
C29	0.5418 (3)	0.7806 (3)	0.45937 (16)	0.0768 (10)	
H29	0.569183	0.795759	0.522546	0.092*	
C30	0.4580 (3)	0.8191 (2)	0.41299 (16)	0.0661 (8)	
H30	0.427801	0.861977	0.444187	0.079*	
C31	0.4166 (2)	0.79596 (18)	0.32057 (14)	0.0469 (5)	
H31	0.357572	0.823171	0.290088	0.056*	
C32	0.35697 (15)	0.56817 (14)	0.13074 (11)	0.0288 (3)	
C33	0.37922 (16)	0.51452 (15)	0.06121 (11)	0.0314 (3)	
H33	0.425126	0.556420	0.028015	0.038*	
C34	0.33679 (17)	0.40263 (16)	0.03904 (12)	0.0357 (4)	
H34	0.355677	0.369708	−0.007685	0.043*	
C35	0.26734 (18)	0.33891 (16)	0.08447 (13)	0.0386 (4)	
H35	0.238500	0.262297	0.069493	0.046*	
C36	0.24016 (18)	0.38826 (16)	0.15238 (13)	0.0375 (4)	
H36	0.190876	0.345394	0.183505	0.045*	
C37	0.28496 (17)	0.49968 (15)	0.17444 (12)	0.0328 (4)	
H37	0.266206	0.531838	0.221614	0.039*	
C38	0.52340 (17)	0.76522 (15)	0.12307 (12)	0.0326 (4)	
C39	0.4939 (2)	0.7860 (2)	0.03918 (15)	0.0483 (5)	
H39	0.403932	0.760101	0.003011	0.058*	
C40	0.5905 (3)	0.8432 (2)	0.00576 (18)	0.0591 (6)	
H40	0.565407	0.857366	−0.051189	0.071*	
C41	0.7210 (3)	0.87880 (19)	0.05451 (18)	0.0572 (7)	
H41	0.787152	0.918291	0.032243	0.069*	
C42	0.7548 (2)	0.85645 (18)	0.13635 (16)	0.0508 (6)	
H42	0.844994	0.878487	0.170137	0.061*	
C43	0.65782 (18)	0.80176 (16)	0.17015 (14)	0.0394 (4)	
H43	0.684101	0.788790	0.227481	0.047*	
C44	0.27982 (17)	0.72875 (15)	0.11957 (11)	0.0320 (4)	
C45	0.14906 (17)	0.65120 (15)	0.07830 (11)	0.0310 (3)	
H45	0.130892	0.576484	0.071996	0.037*	
C46	0.04444 (18)	0.67864 (17)	0.04607 (12)	0.0362 (4)	
H46	−0.042807	0.623166	0.018670	0.043*	
C47	0.0672 (2)	0.78577 (19)	0.05386 (14)	0.0459 (5)	
H47	−0.003866	0.805218	0.033215	0.055*	
C48	0.1950 (2)	0.8647 (2)	0.09213 (18)	0.0561 (6)	
H48	0.212405	0.939090	0.096583	0.067*	
C49	0.2982 (2)	0.83617 (18)	0.12416 (16)	0.0478 (5)	
H49	0.385157	0.892280	0.150376	0.057*	
B1	0.40542 (18)	0.69972 (16)	0.16043 (13)	0.0297 (4)	

### Di-µ-acetato-κ4O:O'-bis{[2-methoxy-N,N-bis(quinolin-2-ylmethyl)ethanamine-κ4N,N',N'',O]manganese(II)} bis(tetraphenylborate) dichloromethane 1.45-solvate (1) Atomic displacement parameters (Å^2^)

**Table:**

	*U*^11^	*U*^22^	*U*^33^	*U*^12^	*U*^13^	*U*^23^
C50	0.078 (3)	0.088 (4)	0.095 (4)	0.034 (3)	0.033 (3)	0.019 (3)
Cl1	0.0627 (15)	0.0701 (19)	0.124 (3)	0.0262 (11)	0.0198 (15)	0.0036 (17)
Cl2	0.0863 (15)	0.128 (2)	0.0717 (14)	0.0313 (14)	0.0241 (11)	0.0371 (13)
C50B	0.097 (5)	0.097 (4)	0.112 (4)	0.028 (4)	0.030 (4)	0.006 (4)
Cl1B	0.107 (4)	0.061 (3)	0.111 (4)	−0.004 (3)	0.032 (4)	0.030 (3)
Cl2B	0.107 (4)	0.121 (3)	0.101 (3)	0.037 (3)	0.016 (3)	−0.008 (3)
C50C	0.085 (5)	0.082 (5)	0.109 (5)	0.024 (4)	0.029 (5)	0.014 (4)
Cl1C	0.106 (5)	0.088 (5)	0.132 (6)	0.016 (5)	0.024 (5)	0.024 (5)
Cl2C	0.086 (5)	0.113 (5)	0.106 (5)	0.024 (4)	0.019 (5)	0.007 (4)
Mn1	0.02674 (13)	0.03049 (14)	0.02112 (11)	0.01047 (10)	0.00803 (9)	0.00420 (9)
O1	0.0261 (6)	0.0485 (8)	0.0334 (6)	0.0143 (5)	0.0069 (5)	0.0116 (6)
O2	0.0464 (8)	0.0493 (8)	0.0283 (6)	0.0213 (6)	0.0187 (5)	0.0123 (6)
O3	0.0324 (7)	0.0494 (9)	0.0538 (9)	0.0165 (6)	0.0012 (6)	0.0164 (7)
N1	0.0311 (7)	0.0276 (7)	0.0225 (6)	0.0118 (6)	0.0078 (5)	0.0045 (5)
N2	0.0310 (7)	0.0294 (7)	0.0250 (6)	0.0102 (6)	0.0100 (5)	0.0069 (5)
N3	0.0338 (8)	0.0304 (7)	0.0296 (7)	0.0127 (6)	0.0075 (6)	0.0024 (6)
C1	0.0307 (8)	0.0269 (8)	0.0264 (7)	0.0117 (6)	0.0072 (6)	0.0067 (6)
C2	0.0596 (13)	0.0352 (10)	0.0361 (10)	0.0150 (9)	0.0228 (9)	0.0103 (8)
C3	0.0804 (17)	0.0434 (13)	0.0549 (13)	0.0177 (12)	0.0380 (13)	0.0223 (11)
C4	0.0716 (16)	0.0319 (11)	0.0660 (16)	0.0092 (11)	0.0262 (13)	0.0184 (11)
C5	0.0571 (13)	0.0289 (10)	0.0527 (12)	0.0123 (9)	0.0136 (10)	0.0054 (9)
C6	0.0392 (9)	0.0279 (9)	0.0327 (8)	0.0136 (7)	0.0063 (7)	0.0033 (7)
C7	0.0601 (13)	0.0333 (10)	0.0337 (9)	0.0199 (9)	0.0143 (9)	−0.0009 (8)
C8	0.0560 (12)	0.0396 (10)	0.0279 (8)	0.0197 (9)	0.0184 (8)	0.0032 (7)
C9	0.0312 (8)	0.0301 (8)	0.0247 (7)	0.0122 (7)	0.0095 (6)	0.0039 (6)
C10	0.0430 (10)	0.0327 (9)	0.0316 (8)	0.0103 (8)	0.0211 (7)	0.0056 (7)
C11	0.0369 (9)	0.0313 (9)	0.0360 (9)	0.0102 (7)	0.0182 (7)	0.0081 (7)
C12	0.0351 (9)	0.0296 (9)	0.0405 (9)	0.0127 (7)	0.0129 (7)	0.0077 (7)
C13	0.0598 (14)	0.0322 (11)	0.0743 (16)	0.0143 (10)	0.0361 (12)	0.0118 (10)
C14	0.0629 (16)	0.0303 (11)	0.092 (2)	0.0108 (10)	0.0335 (14)	0.0019 (12)
C15	0.0491 (12)	0.0364 (11)	0.0526 (12)	0.0182 (9)	0.0080 (10)	−0.0055 (9)
C16	0.0658 (16)	0.0471 (14)	0.0671 (16)	0.0205 (12)	0.0117 (13)	−0.0178 (12)
C17	0.0785 (19)	0.0614 (17)	0.0534 (15)	0.0309 (15)	0.0084 (13)	−0.0219 (13)
C18	0.0874 (19)	0.0707 (18)	0.0417 (12)	0.0395 (15)	0.0241 (12)	−0.0015 (12)
C19	0.0639 (14)	0.0480 (13)	0.0367 (10)	0.0239 (11)	0.0181 (10)	−0.0008 (9)
C20	0.0375 (10)	0.0362 (10)	0.0346 (9)	0.0178 (8)	0.0044 (7)	−0.0021 (7)
C21	0.0409 (10)	0.0527 (12)	0.0297 (8)	0.0220 (9)	0.0089 (7)	0.0177 (8)
C22	0.0360 (10)	0.0578 (13)	0.0275 (8)	0.0203 (9)	0.0018 (7)	0.0101 (8)
C23	0.0256 (9)	0.0617 (14)	0.0590 (13)	0.0137 (9)	0.0114 (9)	0.0214 (11)
C24	0.0287 (8)	0.0337 (9)	0.0257 (7)	0.0100 (7)	0.0099 (6)	0.0036 (6)
C25	0.096 (2)	0.105 (2)	0.0677 (16)	0.0679 (19)	0.0631 (16)	0.0456 (16)
C26	0.0290 (8)	0.0346 (9)	0.0313 (8)	0.0038 (7)	0.0086 (7)	0.0033 (7)
C27	0.0388 (11)	0.0522 (13)	0.0375 (10)	0.0095 (9)	0.0037 (8)	0.0105 (9)
C28	0.0485 (13)	0.0771 (19)	0.0418 (12)	0.0015 (12)	−0.0022 (10)	0.0218 (12)
C29	0.0674 (17)	0.085 (2)	0.0291 (11)	−0.0109 (15)	0.0112 (11)	0.0026 (12)
C30	0.0644 (16)	0.0672 (17)	0.0406 (12)	−0.0006 (13)	0.0277 (11)	−0.0048 (11)
C31	0.0433 (11)	0.0459 (12)	0.0386 (10)	0.0044 (9)	0.0201 (8)	−0.0006 (9)
C32	0.0227 (7)	0.0334 (9)	0.0277 (7)	0.0117 (6)	0.0061 (6)	0.0054 (6)
C33	0.0256 (8)	0.0365 (9)	0.0295 (8)	0.0123 (7)	0.0083 (6)	0.0047 (7)
C34	0.0298 (8)	0.0392 (10)	0.0343 (9)	0.0157 (7)	0.0078 (7)	0.0000 (7)
C35	0.0327 (9)	0.0326 (9)	0.0450 (10)	0.0138 (7)	0.0077 (8)	0.0050 (8)
C36	0.0324 (9)	0.0377 (10)	0.0416 (10)	0.0128 (8)	0.0130 (7)	0.0139 (8)
C37	0.0294 (8)	0.0368 (9)	0.0318 (8)	0.0137 (7)	0.0111 (7)	0.0075 (7)
C38	0.0325 (9)	0.0290 (8)	0.0371 (9)	0.0127 (7)	0.0154 (7)	0.0050 (7)
C39	0.0480 (12)	0.0599 (14)	0.0465 (11)	0.0256 (11)	0.0228 (9)	0.0220 (10)
C40	0.0778 (18)	0.0622 (16)	0.0590 (14)	0.0343 (14)	0.0430 (13)	0.0305 (12)
C41	0.0621 (15)	0.0371 (11)	0.0750 (17)	0.0094 (10)	0.0476 (13)	0.0059 (11)
C42	0.0373 (11)	0.0403 (11)	0.0603 (14)	0.0024 (8)	0.0244 (9)	−0.0090 (10)
C43	0.0338 (9)	0.0359 (10)	0.0403 (10)	0.0088 (7)	0.0143 (7)	−0.0021 (8)
C44	0.0318 (8)	0.0348 (9)	0.0290 (8)	0.0143 (7)	0.0117 (6)	0.0037 (7)
C45	0.0323 (8)	0.0367 (9)	0.0255 (7)	0.0144 (7)	0.0126 (6)	0.0079 (7)
C46	0.0309 (9)	0.0511 (11)	0.0275 (8)	0.0182 (8)	0.0108 (7)	0.0094 (7)
C47	0.0444 (11)	0.0552 (13)	0.0445 (11)	0.0316 (10)	0.0113 (9)	0.0095 (9)
C48	0.0547 (13)	0.0394 (12)	0.0707 (16)	0.0277 (10)	0.0095 (11)	0.0045 (11)
C49	0.0387 (11)	0.0354 (11)	0.0585 (13)	0.0148 (8)	0.0067 (9)	0.0006 (9)
B1	0.0269 (9)	0.0312 (9)	0.0282 (8)	0.0112 (7)	0.0087 (7)	0.0040 (7)

### Di-µ-acetato-κ4O:O'-bis{[2-methoxy-N,N-bis(quinolin-2-ylmethyl)ethanamine-κ4N,N',N'',O]manganese(II)} bis(tetraphenylborate) dichloromethane 1.45-solvate (1) Geometric parameters (Å, º)

**Table:**

C50—H50A	0.9900	C19—H19	0.9500
C50—H50B	0.9900	C19—C20	1.395 (3)
C50—Cl1	1.759 (9)	C21—H21A	0.9900
C50—Cl2	1.690 (9)	C21—H21B	0.9900
C50B—H50C	0.9900	C21—C22	1.504 (3)
C50B—H50D	0.9900	C22—H22A	0.9900
C50B—Cl1B	1.705 (12)	C22—H22B	0.9900
C50B—Cl2B	1.694 (12)	C23—H23A	0.9800
C50C—H50E	0.9900	C23—H23B	0.9800
C50C—H50F	0.9900	C23—H23C	0.9800
C50C—Cl1C	1.727 (16)	C24—C25	1.498 (3)
C50C—Cl2C	1.744 (16)	C25—H25A	0.9800
Mn1—O1	2.3225 (12)	C25—H25B	0.9800
Mn1—O2	2.0617 (13)	C25—H25C	0.9800
Mn1—O3^i^	2.0908 (14)	C26—C27	1.403 (3)
Mn1—N1	2.3179 (14)	C26—C31	1.396 (3)
Mn1—N2	2.2730 (14)	C26—B1	1.653 (3)
Mn1—N3	2.3588 (16)	C27—H27	0.9500
O1—C22	1.428 (2)	C27—C28	1.395 (3)
O1—C23	1.433 (2)	C28—H28	0.9500
O2—C24	1.258 (2)	C28—C29	1.376 (5)
O3—C24	1.229 (2)	C29—H29	0.9500
N1—C1	1.372 (2)	C29—C30	1.366 (5)
N1—C9	1.320 (2)	C30—H30	0.9500
N2—C10	1.471 (2)	C30—C31	1.389 (3)
N2—C11	1.466 (2)	C31—H31	0.9500
N2—C21	1.481 (2)	C32—C33	1.403 (2)
N3—C12	1.321 (2)	C32—C37	1.405 (2)
N3—C20	1.375 (2)	C32—B1	1.636 (3)
C1—C2	1.405 (3)	C33—H33	0.9500
C1—C6	1.413 (2)	C33—C34	1.387 (3)
C2—H2	0.9500	C34—H34	0.9500
C2—C3	1.365 (3)	C34—C35	1.379 (3)
C3—H3	0.9500	C35—H35	0.9500
C3—C4	1.402 (3)	C35—C36	1.389 (3)
C4—H4	0.9500	C36—H36	0.9500
C4—C5	1.350 (4)	C36—C37	1.378 (3)
C5—H5	0.9500	C37—H37	0.9500
C5—C6	1.413 (3)	C38—C39	1.391 (3)
C6—C7	1.407 (3)	C38—C43	1.397 (3)
C7—H7	0.9500	C38—B1	1.649 (3)
C7—C8	1.352 (3)	C39—H39	0.9500
C8—H8	0.9500	C39—C40	1.395 (3)
C8—C9	1.413 (2)	C40—H40	0.9500
C9—C10	1.507 (3)	C40—C41	1.366 (4)
C10—H10A	0.9900	C41—H41	0.9500
C10—H10B	0.9900	C41—C42	1.374 (4)
C11—H11A	0.9900	C42—H42	0.9500
C11—H11B	0.9900	C42—C43	1.392 (3)
C11—C12	1.503 (3)	C43—H43	0.9500
C12—C13	1.409 (3)	C44—C45	1.400 (2)
C13—H13	0.9500	C44—C49	1.394 (3)
C13—C14	1.355 (3)	C44—B1	1.643 (3)
C14—H14	0.9500	C45—H45	0.9500
C14—C15	1.391 (4)	C45—C46	1.392 (3)
C15—C16	1.416 (3)	C46—H46	0.9500
C15—C20	1.423 (3)	C46—C47	1.372 (3)
C16—H16	0.9500	C47—H47	0.9500
C16—C17	1.342 (4)	C47—C48	1.378 (3)
C17—H17	0.9500	C48—H48	0.9500
C17—C18	1.401 (4)	C48—C49	1.386 (3)
C18—H18	0.9500	C49—H49	0.9500
C18—C19	1.377 (3)		
			
H50A—C50—H50B	108.1	C18—C19—C20	120.2 (2)
Cl1—C50—H50A	109.6	C20—C19—H19	119.9
Cl1—C50—H50B	109.6	N3—C20—C15	121.25 (19)
Cl2—C50—H50A	109.6	N3—C20—C19	119.40 (19)
Cl2—C50—H50B	109.6	C19—C20—C15	119.34 (19)
Cl2—C50—Cl1	110.3 (6)	N2—C21—H21A	109.2
H50C—C50B—H50D	105.2	N2—C21—H21B	109.2
Cl1B—C50B—H50C	103.3	N2—C21—C22	112.12 (16)
Cl1B—C50B—H50D	103.3	H21A—C21—H21B	107.9
Cl2B—C50B—H50C	103.3	C22—C21—H21A	109.2
Cl2B—C50B—H50D	103.3	C22—C21—H21B	109.2
Cl2B—C50B—Cl1B	135.4 (13)	O1—C22—C21	107.05 (15)
H50E—C50C—H50F	107.9	O1—C22—H22A	110.3
Cl1C—C50C—H50E	109.2	O1—C22—H22B	110.3
Cl1C—C50C—H50F	109.2	C21—C22—H22A	110.3
Cl1C—C50C—Cl2C	112.2 (17)	C21—C22—H22B	110.3
Cl2C—C50C—H50E	109.2	H22A—C22—H22B	108.6
Cl2C—C50C—H50F	109.2	O1—C23—H23A	109.5
O1—Mn1—N3	96.57 (5)	O1—C23—H23B	109.5
O2—Mn1—O1	84.05 (5)	O1—C23—H23C	109.5
O2—Mn1—O3^i^	110.97 (6)	H23A—C23—H23B	109.5
O2—Mn1—N1	109.12 (6)	H23A—C23—H23C	109.5
O2—Mn1—N3	101.83 (6)	H23B—C23—H23C	109.5
O3^i^—Mn1—N1	87.99 (6)	O2—C24—C25	116.91 (18)
O3^i^—Mn1—N2	90.53 (6)	O3—C24—O2	125.16 (17)
O3^i^—Mn1—N3	87.05 (6)	O3—C24—C25	117.92 (19)
N1—Mn1—O1	80.52 (5)	C24—C25—H25A	109.5
N2—Mn1—N3	73.25 (5)	C24—C25—H25B	109.5
N2—Mn1—N1	75.56 (5)	C24—C25—H25C	109.5
N1—Mn1—N3	148.35 (5)	H25A—C25—H25B	109.5
N2—Mn1—O1	75.32 (5)	H25A—C25—H25C	109.5
O2—Mn1—N2	157.89 (6)	H25B—C25—H25C	109.5
O3^i^—Mn1—O1	163.58 (6)	C27—C26—B1	120.56 (18)
C22—O1—Mn1	112.30 (11)	C31—C26—C27	115.02 (19)
C22—O1—C23	111.21 (16)	C31—C26—B1	124.32 (18)
C23—O1—Mn1	121.88 (12)	C26—C27—H27	118.8
C24—O2—Mn1	142.36 (13)	C28—C27—C26	122.4 (3)
C24—O3—Mn1^i^	151.82 (14)	C28—C27—H27	118.8
C1—N1—Mn1	128.22 (11)	C27—C28—H28	119.9
C9—N1—Mn1	113.65 (11)	C29—C28—C27	120.2 (3)
C9—N1—C1	118.04 (14)	C29—C28—H28	119.9
C10—N2—Mn1	109.92 (11)	C28—C29—H29	120.4
C10—N2—C21	111.94 (15)	C30—C29—C28	119.2 (2)
C11—N2—Mn1	107.45 (10)	C30—C29—H29	120.4
C11—N2—C10	110.41 (14)	C29—C30—H30	119.7
C11—N2—C21	109.22 (15)	C29—C30—C31	120.5 (3)
C21—N2—Mn1	107.76 (11)	C31—C30—H30	119.7
C12—N3—Mn1	111.67 (12)	C26—C31—H31	118.6
C12—N3—C20	118.07 (17)	C30—C31—C26	122.8 (3)
C20—N3—Mn1	129.60 (13)	C30—C31—H31	118.6
N1—C1—C2	119.51 (16)	C33—C32—C37	114.90 (16)
N1—C1—C6	122.12 (16)	C33—C32—B1	124.78 (16)
C2—C1—C6	118.37 (17)	C37—C32—B1	120.30 (15)
C1—C2—H2	119.8	C32—C33—H33	118.7
C3—C2—C1	120.5 (2)	C34—C33—C32	122.53 (17)
C3—C2—H2	119.8	C34—C33—H33	118.7
C2—C3—H3	119.6	C33—C34—H34	119.8
C2—C3—C4	120.9 (2)	C35—C34—C33	120.37 (17)
C4—C3—H3	119.6	C35—C34—H34	119.8
C3—C4—H4	119.9	C34—C35—H35	120.5
C5—C4—C3	120.1 (2)	C34—C35—C36	119.09 (18)
C5—C4—H4	119.9	C36—C35—H35	120.5
C4—C5—H5	119.8	C35—C36—H36	120.1
C4—C5—C6	120.5 (2)	C37—C36—C35	119.71 (18)
C6—C5—H5	119.8	C37—C36—H36	120.1
C5—C6—C1	119.66 (18)	C32—C37—H37	118.3
C7—C6—C1	117.70 (17)	C36—C37—C32	123.36 (17)
C7—C6—C5	122.64 (18)	C36—C37—H37	118.3
C6—C7—H7	120.2	C39—C38—C43	114.79 (18)
C8—C7—C6	119.61 (17)	C39—C38—B1	121.06 (17)
C8—C7—H7	120.2	C43—C38—B1	124.13 (17)
C7—C8—H8	120.2	C38—C39—H39	118.5
C7—C8—C9	119.59 (18)	C38—C39—C40	123.0 (2)
C9—C8—H8	120.2	C40—C39—H39	118.5
N1—C9—C8	122.90 (17)	C39—C40—H40	119.8
N1—C9—C10	119.42 (15)	C41—C40—C39	120.3 (2)
C8—C9—C10	117.64 (16)	C41—C40—H40	119.8
N2—C10—C9	114.35 (14)	C40—C41—H41	120.6
N2—C10—H10A	108.7	C40—C41—C42	118.7 (2)
N2—C10—H10B	108.7	C42—C41—H41	120.6
C9—C10—H10A	108.7	C41—C42—H42	119.7
C9—C10—H10B	108.7	C41—C42—C43	120.5 (2)
H10A—C10—H10B	107.6	C43—C42—H42	119.7
N2—C11—H11A	109.2	C38—C43—H43	118.7
N2—C11—H11B	109.2	C42—C43—C38	122.6 (2)
N2—C11—C12	112.10 (15)	C42—C43—H43	118.7
H11A—C11—H11B	107.9	C45—C44—B1	124.36 (16)
C12—C11—H11A	109.2	C49—C44—C45	114.81 (17)
C12—C11—H11B	109.2	C49—C44—B1	120.83 (16)
N3—C12—C11	119.00 (16)	C44—C45—H45	118.5
N3—C12—C13	123.29 (18)	C46—C45—C44	122.94 (18)
C13—C12—C11	117.66 (18)	C46—C45—H45	118.5
C12—C13—H13	120.4	C45—C46—H46	120.0
C14—C13—C12	119.1 (2)	C47—C46—C45	120.04 (18)
C14—C13—H13	120.4	C47—C46—H46	120.0
C13—C14—H14	120.1	C46—C47—H47	120.5
C13—C14—C15	119.9 (2)	C46—C47—C48	118.94 (19)
C15—C14—H14	120.1	C48—C47—H47	120.5
C14—C15—C16	123.2 (2)	C47—C48—H48	119.8
C14—C15—C20	118.2 (2)	C47—C48—C49	120.4 (2)
C16—C15—C20	118.5 (2)	C49—C48—H48	119.8
C15—C16—H16	119.6	C44—C49—H49	118.6
C17—C16—C15	120.8 (3)	C48—C49—C44	122.9 (2)
C17—C16—H16	119.6	C48—C49—H49	118.6
C16—C17—H17	119.6	C32—B1—C26	106.64 (15)
C16—C17—C18	120.9 (2)	C32—B1—C38	111.30 (14)
C18—C17—H17	119.6	C32—B1—C44	109.25 (14)
C17—C18—H18	119.9	C38—B1—C26	110.86 (14)
C19—C18—C17	120.3 (3)	C44—B1—C26	110.05 (14)
C19—C18—H18	119.9	C44—B1—C38	108.71 (15)
C18—C19—H19	119.9		
			
Mn1—O1—C22—C21	38.51 (19)	C20—N3—C12—C11	−175.83 (16)
Mn1—O2—C24—O3	10.6 (3)	C20—N3—C12—C13	1.6 (3)
Mn1—O2—C24—C25	−170.5 (2)	C20—C15—C16—C17	0.1 (4)
Mn1^i^—O3—C24—O2	−52.6 (4)	C21—N2—C10—C9	90.09 (19)
Mn1^i^—O3—C24—C25	128.6 (3)	C21—N2—C11—C12	−73.32 (19)
Mn1—N1—C1—C2	3.7 (2)	C23—O1—C22—C21	178.99 (18)
Mn1—N1—C1—C6	−176.51 (12)	C26—C27—C28—C29	0.0 (4)
Mn1—N1—C9—C8	178.81 (14)	C27—C26—C31—C30	0.3 (3)
Mn1—N1—C9—C10	−3.4 (2)	C27—C26—B1—C32	47.7 (2)
Mn1—N2—C10—C9	−29.63 (18)	C27—C26—B1—C38	−73.6 (2)
Mn1—N2—C11—C12	43.31 (17)	C27—C26—B1—C44	166.12 (17)
Mn1—N2—C21—C22	46.04 (18)	C27—C28—C29—C30	−0.4 (4)
Mn1—N3—C12—C11	−4.3 (2)	C28—C29—C30—C31	0.7 (4)
Mn1—N3—C12—C13	173.17 (18)	C29—C30—C31—C26	−0.7 (4)
Mn1—N3—C20—C15	−167.79 (15)	C31—C26—C27—C28	0.0 (3)
Mn1—N3—C20—C19	10.9 (3)	C31—C26—B1—C32	−128.48 (19)
N1—C1—C2—C3	−178.6 (2)	C31—C26—B1—C38	110.2 (2)
N1—C1—C6—C5	178.65 (18)	C31—C26—B1—C44	−10.1 (2)
N1—C1—C6—C7	−1.4 (3)	C32—C33—C34—C35	1.4 (3)
N1—C9—C10—N2	23.0 (2)	C33—C32—C37—C36	0.8 (2)
N2—C11—C12—N3	−26.8 (2)	C33—C32—B1—C26	−139.84 (16)
N2—C11—C12—C13	155.63 (19)	C33—C32—B1—C38	−18.8 (2)
N2—C21—C22—O1	−57.5 (2)	C33—C32—B1—C44	101.24 (18)
N3—C12—C13—C14	−3.4 (4)	C33—C34—C35—C36	0.2 (3)
C1—N1—C9—C8	1.9 (3)	C34—C35—C36—C37	−1.3 (3)
C1—N1—C9—C10	179.71 (16)	C35—C36—C37—C32	0.8 (3)
C1—C2—C3—C4	−0.8 (4)	C37—C32—C33—C34	−1.8 (2)
C1—C6—C7—C8	1.3 (3)	C37—C32—B1—C26	41.6 (2)
C2—C1—C6—C5	−1.6 (3)	C37—C32—B1—C38	162.65 (15)
C2—C1—C6—C7	178.30 (19)	C37—C32—B1—C44	−77.29 (19)
C2—C3—C4—C5	−0.1 (4)	C38—C39—C40—C41	2.1 (4)
C3—C4—C5—C6	0.1 (4)	C39—C38—C43—C42	1.1 (3)
C4—C5—C6—C1	0.8 (3)	C39—C38—B1—C26	−153.23 (18)
C4—C5—C6—C7	−179.1 (2)	C39—C38—B1—C32	88.2 (2)
C5—C6—C7—C8	−178.8 (2)	C39—C38—B1—C44	−32.1 (2)
C6—C1—C2—C3	1.6 (3)	C39—C40—C41—C42	0.5 (4)
C6—C7—C8—C9	0.4 (3)	C40—C41—C42—C43	−2.2 (3)
C7—C8—C9—N1	−2.1 (3)	C41—C42—C43—C38	1.4 (3)
C7—C8—C9—C10	−179.91 (19)	C43—C38—C39—C40	−2.8 (3)
C8—C9—C10—N2	−159.09 (17)	C43—C38—B1—C26	28.2 (2)
C9—N1—C1—C2	−179.88 (17)	C43—C38—B1—C32	−90.3 (2)
C9—N1—C1—C6	−0.1 (2)	C43—C38—B1—C44	149.32 (17)
C10—N2—C11—C12	163.18 (16)	C44—C45—C46—C47	0.2 (3)
C10—N2—C21—C22	−74.9 (2)	C45—C44—C49—C48	1.3 (3)
C11—N2—C10—C9	−148.00 (16)	C45—C44—B1—C26	−107.56 (19)
C11—N2—C21—C22	162.47 (16)	C45—C44—B1—C32	9.2 (2)
C11—C12—C13—C14	174.1 (2)	C45—C44—B1—C38	130.85 (17)
C12—N3—C20—C15	2.0 (3)	C45—C46—C47—C48	1.4 (3)
C12—N3—C20—C19	−179.26 (19)	C46—C47—C48—C49	−1.6 (4)
C12—C13—C14—C15	1.4 (4)	C47—C48—C49—C44	0.2 (4)
C13—C14—C15—C16	−178.7 (3)	C49—C44—C45—C46	−1.5 (3)
C13—C14—C15—C20	2.0 (4)	C49—C44—B1—C26	72.5 (2)
C14—C15—C16—C17	−179.2 (3)	C49—C44—B1—C32	−170.74 (18)
C14—C15—C20—N3	−3.8 (3)	C49—C44—B1—C38	−49.1 (2)
C14—C15—C20—C19	177.5 (2)	B1—C26—C27—C28	−176.5 (2)
C15—C16—C17—C18	1.2 (5)	B1—C26—C31—C30	176.7 (2)
C16—C15—C20—N3	176.9 (2)	B1—C32—C33—C34	179.55 (16)
C16—C15—C20—C19	−1.9 (3)	B1—C32—C37—C36	179.43 (16)
C16—C17—C18—C19	−0.7 (5)	B1—C38—C39—C40	178.5 (2)
C17—C18—C19—C20	−1.1 (4)	B1—C38—C43—C42	179.73 (18)
C18—C19—C20—N3	−176.4 (2)	B1—C44—C45—C46	178.56 (16)
C18—C19—C20—C15	2.3 (3)	B1—C44—C49—C48	−178.8 (2)

Symmetry code: (i) −*x*, −*y*+1, −*z*+1.

### Di-µ-acetato-κ4O:O'-bis{[2-methoxy-N,N-bis(quinolin-2-ylmethyl)ethanamine-κ4N,N',N'',O]manganese(II)} bis(tetraphenylborate) dichloromethane 1.45-solvate (1) Hydrogen-bond geometry (Å, º)

*Cg*9 and *Cg*12 are the centroids of the C32–C37 and C44–C49 rings, respectively.

**Table:**

*D*—H···*A*	*D*—H	H···*A*	*D*···*A*	*D*—H···*A*
C2—H2···O2	0.95	2.49	3.366 (3)	154
C19—H19···O2	0.95	2.31	3.199 (3)	155
C23—H23*A*···O2	0.98	2.31	3.1767 (2)	119
C29—H29···Cl2^ii^	0.95	2.65	3.5305 (2)	155
C8—H8···*Cg*11^iii^	0.95	2.68	3.5556 (2)	153
C11—H11*B*···*Cg*11^iv^	0.99	2.81	3.7195 (2)	152
C23—H23*B*···*Cg*9	0.98	2.78	3.7034 (2)	157

Symmetry codes: (ii) −*x*+1, −*y*+1, −*z*+1; (iii) −*x*+1, −*y*+1, −*z*; (iv) *x*+1, *y*, *z*.

### (Acetato-κO)[2-hydroxy-N,N-bis(quinolin-2-ylmethyl)ethanamine-κ4N,N',N'',O](methanol-κO)manganese(II) tetraphenylborate methanol monosolvate (2) Crystal data

**Table:**

[Mn(C_2_H_3_O_2_)(C_22_H_21_N_3_O)(CH_4_O)](C_24_H_20_B)·CH_4_O	*F*(000) = 1772
*M**_r_* = 840.69	*D*_x_ = 1.295 Mg m^−^^3^
Monoclinic, *P*2_1_/*c*	Mo *K*α radiation, λ = 0.71073 Å
*a* = 10.3504 (3) Å	Cell parameters from 8395 reflections
*b* = 17.4824 (5) Å	θ = 2.4–32.4°
*c* = 23.9618 (9) Å	µ = 0.36 mm^−^^1^
β = 96.222 (3)°	*T* = 173 K
*V* = 4310.3 (3) Å^3^	Prism, colourless
*Z* = 4	0.34 × 0.28 × 0.26 mm

### (Acetato-κO)[2-hydroxy-N,N-bis(quinolin-2-ylmethyl)ethanamine-κ4N,N',N'',O](methanol-κO)manganese(II) tetraphenylborate methanol monosolvate (2) Data collection

**Table:**

Rigaku Oxford Diffraction Gemini Eos diffractometer	14443 independent reflections
Radiation source: fine-focus sealed X-ray tube	10348 reflections with *I* > 2σ(*I*)
Detector resolution: 16.0416 pixels mm^-1^	*R*_int_ = 0.032
ω scans	θ_max_ = 32.9°, θ_min_ = 2.4°
Absorption correction: multi-scan (CrysAlisPro; Rigaku OD, 2019)	*h* = −15→12
*T*_min_ = 0.845, *T*_max_ = 1.000	*k* = −25→15
31473 measured reflections	*l* = −34→36

### (Acetato-κO)[2-hydroxy-N,N-bis(quinolin-2-ylmethyl)ethanamine-κ4N,N',N'',O](methanol-κO)manganese(II) tetraphenylborate methanol monosolvate (2) Refinement

**Table:**

Refinement on *F*^2^	Primary atom site location: dual
Least-squares matrix: full	Hydrogen site location: mixed
*R*[*F*^2^ > 2σ(*F*^2^)] = 0.054	H atoms treated by a mixture of independent and constrained refinement
*wR*(*F*^2^) = 0.149	*w* = 1/[σ^2^(*F*_o_^2^) + (0.0597*P*)^2^ + 1.7892*P*] where *P* = (*F*_o_^2^ + 2*F*_c_^2^)/3
*S* = 1.04	(Δ/σ)_max_ = 0.001
14443 reflections	Δρ_max_ = 0.67 e Å^−^^3^
582 parameters	Δρ_min_ = −0.44 e Å^−^^3^
85 restraints	

### (Acetato-κO)[2-hydroxy-N,N-bis(quinolin-2-ylmethyl)ethanamine-κ4N,N',N'',O](methanol-κO)manganese(II) tetraphenylborate methanol monosolvate (2) Special details

**Table:**

Geometry. All esds (except the esd in the dihedral angle between two l.s. planes) are estimated using the full covariance matrix. The cell esds are taken into account individually in the estimation of esds in distances, angles and torsion angles; correlations between esds in cell parameters are only used when they are defined by crystal symmetry. An approximate (isotropic) treatment of cell esds is used for estimating esds involving l.s. planes.

### (Acetato-κO)[2-hydroxy-N,N-bis(quinolin-2-ylmethyl)ethanamine-κ4N,N',N'',O](methanol-κO)manganese(II) tetraphenylborate methanol monosolvate (2) Fractional atomic coordinates and isotropic or equivalent isotropic displacement parameters (Å^2^)

**Table:**

	*x*	*y*	*z*	*U*_iso_*/*U*_eq_	Occ. (<1)
Mn1	0.20955 (3)	0.33373 (2)	0.52463 (2)	0.02919 (8)	
O1	0.28258 (14)	0.44314 (8)	0.52710 (7)	0.0462 (4)	
O2	0.13629 (13)	0.53266 (9)	0.50981 (7)	0.0474 (4)	
C21	−0.0123 (4)	0.2185 (2)	0.51942 (16)	0.0392 (8)	0.791 (5)
H21A	−0.046372	0.236717	0.554101	0.047*	0.791 (5)
H21B	−0.050269	0.167489	0.510100	0.047*	0.791 (5)
C22	−0.0519 (2)	0.27345 (14)	0.47199 (13)	0.0418 (7)	0.791 (5)
H22A	−0.026426	0.252695	0.436329	0.050*	0.791 (5)
H22B	−0.147385	0.280376	0.467818	0.050*	0.791 (5)
O3	0.0113 (7)	0.3457 (3)	0.4842 (2)	0.0403 (9)	0.791 (5)
H3	−0.040 (3)	0.3839 (18)	0.4820 (17)	0.060*	0.791 (5)
C21B	−0.0034 (13)	0.2064 (8)	0.5036 (7)	0.037 (2)	0.209 (5)
H21C	−0.047254	0.162125	0.519187	0.044*	0.209 (5)
H21D	−0.006777	0.200111	0.462437	0.044*	0.209 (5)
C22B	−0.0669 (8)	0.2791 (5)	0.5177 (5)	0.0389 (19)	0.209 (5)
H22C	−0.157693	0.280497	0.499824	0.047*	0.209 (5)
H22D	−0.067722	0.283649	0.558803	0.047*	0.209 (5)
O3B	0.008 (3)	0.3419 (13)	0.4966 (10)	0.043 (3)	0.209 (5)
H3B	−0.056 (11)	0.371 (8)	0.499 (7)	0.065*	0.209 (5)
O4	0.11509 (17)	0.35761 (10)	0.60633 (7)	0.0527 (4)	
H4	0.055 (2)	0.3937 (15)	0.5997 (13)	0.079*	
N1	0.13266 (14)	0.21202 (8)	0.52926 (7)	0.0303 (3)	
N2	0.25628 (13)	0.27746 (8)	0.44162 (6)	0.0266 (3)	
N3	0.35823 (13)	0.26902 (8)	0.58298 (6)	0.0252 (3)	
C1	0.1944 (2)	0.16292 (10)	0.48974 (8)	0.0357 (4)	
H1A	0.133326	0.121192	0.477214	0.043*	
H1B	0.272630	0.139148	0.510029	0.043*	
C2	0.23347 (16)	0.20309 (9)	0.43876 (7)	0.0280 (3)	
C3	0.25111 (19)	0.15814 (10)	0.39141 (8)	0.0334 (4)	
H3A	0.233076	0.104858	0.391410	0.040*	
C4	0.29409 (19)	0.19162 (11)	0.34578 (8)	0.0356 (4)	
H4A	0.308919	0.161726	0.313979	0.043*	
C5	0.31655 (16)	0.27117 (10)	0.34599 (7)	0.0283 (3)	
C6	0.35729 (18)	0.31007 (11)	0.29957 (8)	0.0353 (4)	
H6	0.370740	0.282313	0.266608	0.042*	
C7	0.37775 (18)	0.38724 (12)	0.30132 (9)	0.0381 (4)	
H7	0.405897	0.412934	0.269861	0.046*	
C8	0.35708 (19)	0.42820 (11)	0.34959 (9)	0.0393 (4)	
H8	0.370369	0.481974	0.350415	0.047*	
C9	0.31792 (18)	0.39232 (10)	0.39589 (9)	0.0345 (4)	
H9	0.305140	0.421140	0.428451	0.041*	
C10	0.29674 (15)	0.31276 (9)	0.39503 (7)	0.0259 (3)	
C11	0.17185 (17)	0.18520 (10)	0.58677 (8)	0.0312 (4)	
H11A	0.163531	0.128838	0.588150	0.037*	
H11B	0.113287	0.207489	0.612489	0.037*	
C12	0.31033 (16)	0.20774 (9)	0.60597 (7)	0.0267 (3)	
C13	0.38127 (18)	0.16443 (10)	0.64823 (7)	0.0312 (3)	
H13	0.343839	0.120350	0.663192	0.037*	
C14	0.50433 (18)	0.18645 (11)	0.66747 (7)	0.0319 (4)	
H14	0.552399	0.158636	0.696811	0.038*	
C15	0.56008 (16)	0.25053 (10)	0.64375 (7)	0.0276 (3)	
C16	0.68919 (18)	0.27456 (12)	0.66024 (8)	0.0364 (4)	
H16	0.740753	0.248239	0.689357	0.044*	
C17	0.73966 (18)	0.33547 (12)	0.63438 (9)	0.0396 (4)	
H17	0.826480	0.351267	0.645467	0.047*	
C18	0.66421 (17)	0.37488 (11)	0.59156 (9)	0.0356 (4)	
H18	0.700919	0.416920	0.573713	0.043*	
C19	0.53883 (17)	0.35388 (10)	0.57505 (8)	0.0300 (3)	
H19	0.488663	0.381583	0.546259	0.036*	
C20	0.48377 (15)	0.29084 (9)	0.60089 (7)	0.0250 (3)	
C23	0.24089 (16)	0.50955 (10)	0.53307 (8)	0.0303 (3)	
C24	0.3267 (3)	0.56395 (15)	0.56863 (12)	0.0614 (7)	
H24A	0.404881	0.574672	0.550308	0.092*	
H24B	0.351550	0.540939	0.605520	0.092*	
H24C	0.279510	0.611747	0.573258	0.092*	
C25	0.1491 (3)	0.3478 (2)	0.66598 (13)	0.0784 (10)	
H25A	0.236358	0.325640	0.672799	0.118*	
H25B	0.086276	0.313477	0.680887	0.118*	
H25C	0.147710	0.397564	0.684705	0.118*	
C1A	0.22940 (15)	0.82390 (9)	0.66632 (7)	0.0259 (3)	
C2A	0.10210 (17)	0.80890 (11)	0.67793 (9)	0.0355 (4)	
H2A	0.038937	0.848323	0.672024	0.043*	
C3A	0.06469 (19)	0.73829 (13)	0.69782 (10)	0.0447 (5)	
H3AA	−0.022383	0.730787	0.705735	0.054*	
C4A	0.1531 (2)	0.67907 (11)	0.70614 (9)	0.0387 (4)	
H4AA	0.127219	0.630697	0.719175	0.046*	
C5A	0.27959 (18)	0.69140 (10)	0.69519 (8)	0.0326 (4)	
H5A	0.341484	0.651201	0.700378	0.039*	
C6A	0.31643 (16)	0.76255 (10)	0.67659 (8)	0.0297 (3)	
H6A	0.404714	0.770115	0.670519	0.036*	
C7A	0.31146 (15)	0.89862 (10)	0.57761 (7)	0.0261 (3)	
C8A	0.3448 (2)	0.83050 (11)	0.55251 (9)	0.0380 (4)	
H8A	0.343659	0.784419	0.573459	0.046*	
C9A	0.3796 (3)	0.82714 (13)	0.49820 (10)	0.0529 (6)	
H9A	0.401198	0.779232	0.482977	0.063*	
C10A	0.3831 (2)	0.89245 (14)	0.46602 (9)	0.0466 (5)	
H10A	0.406822	0.890104	0.428855	0.056*	
C11A	0.35142 (19)	0.96101 (13)	0.48919 (8)	0.0389 (4)	
H11C	0.353786	1.006842	0.468020	0.047*	
C12A	0.31613 (17)	0.96345 (11)	0.54320 (8)	0.0329 (4)	
H12A	0.293816	1.011597	0.557854	0.040*	
C13A	0.16269 (15)	0.97007 (9)	0.64705 (8)	0.0267 (3)	
C14A	0.05958 (16)	0.97907 (10)	0.60442 (9)	0.0353 (4)	
H14A	0.059256	0.949083	0.571341	0.042*	
C15A	−0.04201 (17)	1.02996 (11)	0.60857 (11)	0.0432 (5)	
H15A	−0.110442	1.033710	0.578894	0.052*	
C16A	−0.04368 (19)	1.07520 (12)	0.65585 (11)	0.0474 (6)	
H16A	−0.112196	1.110670	0.658681	0.057*	
C17A	0.0554 (2)	1.06805 (12)	0.69877 (10)	0.0441 (5)	
H17A	0.055435	1.098602	0.731560	0.053*	
C18A	0.15569 (17)	1.01606 (11)	0.69413 (8)	0.0335 (4)	
H18A	0.222400	1.011741	0.724482	0.040*	
C19A	0.41144 (14)	0.93303 (9)	0.68196 (7)	0.0232 (3)	
C20A	0.51032 (15)	0.97532 (10)	0.66120 (8)	0.0287 (3)	
H20A	0.501709	0.988501	0.622509	0.034*	
C21A	0.62107 (17)	0.99890 (10)	0.69507 (9)	0.0352 (4)	
H21E	0.685705	1.027867	0.679253	0.042*	
C22A	0.63763 (18)	0.98059 (11)	0.75126 (9)	0.0375 (4)	
H22E	0.713240	0.996573	0.774337	0.045*	
C23A	0.54236 (19)	0.93855 (11)	0.77356 (8)	0.0366 (4)	
H23A	0.552187	0.925441	0.812265	0.044*	
C24A	0.43231 (17)	0.91548 (10)	0.73937 (7)	0.0300 (3)	
H24D	0.368273	0.886520	0.755581	0.036*	
B1	0.27814 (16)	0.90606 (10)	0.64275 (8)	0.0242 (3)	
O1S	−0.05332 (15)	0.47197 (9)	0.60054 (7)	0.0472 (4)	
H1S	−0.068772	0.486107	0.566974	0.071*	
C1S	−0.0042 (2)	0.53448 (14)	0.63392 (11)	0.0520 (6)	
H1SA	−0.047010	0.581705	0.619749	0.078*	
H1SB	0.089674	0.538828	0.632230	0.078*	
H1SC	−0.021328	0.526189	0.672897	0.078*	

### (Acetato-κO)[2-hydroxy-N,N-bis(quinolin-2-ylmethyl)ethanamine-κ4N,N',N'',O](methanol-κO)manganese(II) tetraphenylborate methanol monosolvate (2) Atomic displacement parameters (Å^2^)

**Table:**

	*U*^11^	*U*^22^	*U*^33^	*U*^12^	*U*^13^	*U*^23^
Mn1	0.02894 (13)	0.02222 (13)	0.03608 (15)	−0.00182 (9)	0.00209 (10)	−0.00026 (10)
O1	0.0414 (7)	0.0229 (6)	0.0737 (11)	0.0002 (5)	0.0041 (7)	−0.0025 (6)
O2	0.0346 (7)	0.0392 (8)	0.0650 (10)	0.0033 (6)	−0.0096 (7)	−0.0072 (7)
C21	0.0324 (13)	0.0326 (16)	0.051 (2)	−0.0094 (11)	−0.0030 (14)	0.0003 (13)
C22	0.0340 (12)	0.0340 (13)	0.0541 (17)	−0.0049 (9)	−0.0099 (11)	−0.0010 (11)
O3	0.0302 (12)	0.0282 (13)	0.060 (3)	0.0003 (10)	−0.0077 (17)	−0.0041 (14)
C21B	0.029 (4)	0.032 (4)	0.047 (5)	−0.008 (3)	−0.001 (4)	−0.004 (4)
C22B	0.028 (3)	0.032 (3)	0.057 (4)	−0.005 (3)	0.007 (3)	−0.003 (3)
O3B	0.036 (4)	0.033 (4)	0.059 (6)	0.004 (3)	0.000 (5)	0.009 (4)
O4	0.0568 (10)	0.0561 (10)	0.0459 (9)	0.0133 (8)	0.0089 (7)	−0.0098 (7)
N1	0.0296 (7)	0.0264 (7)	0.0346 (8)	−0.0053 (5)	0.0021 (6)	0.0003 (6)
N2	0.0274 (6)	0.0215 (6)	0.0306 (7)	−0.0026 (5)	0.0017 (5)	0.0030 (5)
N3	0.0270 (6)	0.0225 (6)	0.0269 (7)	−0.0020 (5)	0.0071 (5)	0.0009 (5)
C1	0.0546 (11)	0.0213 (8)	0.0311 (9)	−0.0066 (7)	0.0039 (8)	0.0011 (6)
C2	0.0296 (8)	0.0225 (7)	0.0308 (8)	−0.0020 (6)	−0.0011 (6)	0.0012 (6)
C3	0.0442 (10)	0.0214 (8)	0.0341 (9)	−0.0025 (7)	0.0028 (8)	−0.0008 (6)
C4	0.0434 (10)	0.0296 (9)	0.0338 (9)	0.0004 (7)	0.0049 (8)	−0.0026 (7)
C5	0.0252 (7)	0.0271 (8)	0.0324 (9)	0.0023 (6)	0.0020 (6)	0.0035 (6)
C6	0.0360 (9)	0.0365 (10)	0.0340 (9)	0.0030 (7)	0.0070 (7)	0.0048 (7)
C7	0.0360 (9)	0.0384 (10)	0.0408 (11)	0.0013 (8)	0.0074 (8)	0.0119 (8)
C8	0.0428 (10)	0.0273 (9)	0.0488 (12)	−0.0024 (7)	0.0088 (9)	0.0072 (8)
C9	0.0383 (9)	0.0253 (8)	0.0408 (10)	−0.0028 (7)	0.0086 (8)	0.0013 (7)
C10	0.0220 (7)	0.0239 (7)	0.0315 (8)	0.0001 (6)	0.0014 (6)	0.0035 (6)
C11	0.0313 (8)	0.0287 (8)	0.0348 (9)	−0.0063 (6)	0.0092 (7)	0.0016 (7)
C12	0.0292 (8)	0.0248 (8)	0.0274 (8)	−0.0025 (6)	0.0097 (6)	−0.0005 (6)
C13	0.0380 (9)	0.0280 (8)	0.0287 (8)	−0.0022 (7)	0.0085 (7)	0.0041 (6)
C14	0.0376 (9)	0.0323 (9)	0.0260 (8)	0.0025 (7)	0.0040 (7)	0.0046 (6)
C15	0.0295 (8)	0.0273 (8)	0.0269 (8)	0.0024 (6)	0.0069 (6)	−0.0012 (6)
C16	0.0332 (9)	0.0403 (10)	0.0351 (10)	0.0021 (7)	0.0012 (7)	−0.0009 (8)
C17	0.0291 (9)	0.0454 (11)	0.0439 (11)	−0.0068 (8)	0.0032 (8)	−0.0025 (8)
C18	0.0317 (8)	0.0312 (9)	0.0450 (11)	−0.0065 (7)	0.0088 (8)	0.0009 (7)
C19	0.0309 (8)	0.0249 (8)	0.0352 (9)	−0.0017 (6)	0.0073 (7)	0.0022 (6)
C20	0.0253 (7)	0.0218 (7)	0.0289 (8)	0.0003 (6)	0.0077 (6)	−0.0018 (6)
C23	0.0299 (8)	0.0254 (8)	0.0354 (9)	−0.0037 (6)	0.0027 (7)	−0.0017 (6)
C24	0.0570 (14)	0.0509 (14)	0.0712 (18)	−0.0076 (11)	−0.0169 (12)	−0.0204 (12)
C25	0.0734 (19)	0.093 (2)	0.0644 (19)	0.0182 (16)	−0.0132 (15)	−0.0370 (17)
C1A	0.0239 (7)	0.0248 (8)	0.0289 (8)	−0.0040 (6)	0.0030 (6)	−0.0026 (6)
C2A	0.0230 (7)	0.0353 (9)	0.0486 (11)	−0.0038 (7)	0.0051 (7)	0.0046 (8)
C3A	0.0304 (9)	0.0429 (11)	0.0615 (14)	−0.0123 (8)	0.0087 (9)	0.0073 (10)
C4A	0.0440 (10)	0.0297 (9)	0.0420 (11)	−0.0124 (8)	0.0027 (8)	0.0037 (7)
C5A	0.0384 (9)	0.0242 (8)	0.0344 (9)	−0.0020 (7)	−0.0001 (7)	−0.0023 (7)
C6A	0.0259 (7)	0.0253 (8)	0.0379 (9)	−0.0021 (6)	0.0038 (7)	−0.0028 (7)
C7A	0.0205 (7)	0.0277 (8)	0.0301 (8)	−0.0019 (6)	0.0020 (6)	−0.0039 (6)
C8A	0.0437 (10)	0.0296 (9)	0.0438 (11)	−0.0057 (8)	0.0182 (9)	−0.0081 (8)
C9A	0.0690 (15)	0.0421 (12)	0.0532 (14)	−0.0081 (10)	0.0323 (12)	−0.0192 (10)
C10A	0.0506 (12)	0.0585 (14)	0.0329 (10)	−0.0097 (10)	0.0154 (9)	−0.0122 (9)
C11A	0.0372 (9)	0.0489 (12)	0.0302 (9)	0.0006 (8)	0.0024 (7)	0.0031 (8)
C12A	0.0351 (9)	0.0347 (9)	0.0289 (9)	0.0052 (7)	0.0027 (7)	0.0001 (7)
C13A	0.0200 (6)	0.0222 (7)	0.0387 (9)	−0.0020 (5)	0.0075 (6)	−0.0002 (6)
C14A	0.0245 (8)	0.0272 (8)	0.0533 (12)	−0.0023 (6)	−0.0001 (7)	−0.0015 (8)
C15A	0.0201 (8)	0.0336 (10)	0.0754 (15)	−0.0017 (7)	0.0028 (8)	0.0101 (10)
C16A	0.0294 (9)	0.0323 (10)	0.0853 (17)	0.0059 (7)	0.0279 (10)	0.0079 (10)
C17A	0.0428 (10)	0.0351 (10)	0.0598 (13)	0.0029 (8)	0.0296 (10)	−0.0042 (9)
C18A	0.0309 (8)	0.0313 (9)	0.0404 (10)	−0.0003 (7)	0.0132 (7)	−0.0019 (7)
C19A	0.0208 (6)	0.0186 (7)	0.0304 (8)	−0.0006 (5)	0.0041 (6)	−0.0026 (6)
C20A	0.0243 (7)	0.0267 (8)	0.0353 (9)	−0.0032 (6)	0.0036 (6)	0.0037 (6)
C21A	0.0239 (7)	0.0265 (8)	0.0548 (12)	−0.0052 (6)	0.0023 (7)	0.0021 (8)
C22A	0.0300 (8)	0.0280 (9)	0.0512 (12)	0.0005 (7)	−0.0104 (8)	−0.0079 (8)
C23A	0.0415 (10)	0.0341 (10)	0.0325 (9)	0.0007 (8)	−0.0036 (8)	−0.0040 (7)
C24A	0.0310 (8)	0.0292 (8)	0.0303 (8)	−0.0034 (6)	0.0060 (7)	−0.0029 (6)
B1	0.0208 (7)	0.0218 (8)	0.0304 (9)	−0.0016 (6)	0.0045 (6)	−0.0025 (6)
O1S	0.0457 (8)	0.0450 (9)	0.0509 (9)	−0.0040 (7)	0.0050 (7)	−0.0076 (7)
C1S	0.0468 (12)	0.0492 (13)	0.0588 (15)	0.0004 (10)	0.0004 (10)	−0.0151 (11)

### (Acetato-κO)[2-hydroxy-N,N-bis(quinolin-2-ylmethyl)ethanamine-κ4N,N',N'',O](methanol-κO)manganese(II) tetraphenylborate methanol monosolvate (2) Geometric parameters (Å, º)

**Table:**

Mn1—O1	2.0551 (14)	C18—H18	0.9500
Mn1—O3	2.182 (7)	C18—C19	1.365 (2)
Mn1—O3B	2.13 (3)	C19—H19	0.9500
Mn1—O4	2.3190 (16)	C19—C20	1.414 (2)
Mn1—N1	2.2787 (15)	C23—C24	1.501 (3)
Mn1—N2	2.3167 (15)	C24—H24A	0.9800
Mn1—N3	2.2664 (14)	C24—H24B	0.9800
O1—C23	1.252 (2)	C24—H24C	0.9800
O2—C23	1.231 (2)	C25—H25A	0.9800
C21—H21A	0.9900	C25—H25B	0.9800
C21—H21B	0.9900	C25—H25C	0.9800
C21—C22	1.511 (5)	C1A—C2A	1.401 (2)
C21—N1	1.497 (4)	C1A—C6A	1.405 (2)
C22—H22A	0.9900	C1A—B1	1.643 (2)
C22—H22B	0.9900	C2A—H2A	0.9500
C22—O3	1.438 (5)	C2A—C3A	1.393 (3)
O3—H3	0.849 (18)	C3A—H3AA	0.9500
C21B—H21C	0.9900	C3A—C4A	1.382 (3)
C21B—H21D	0.9900	C4A—H4AA	0.9500
C21B—C22B	1.487 (13)	C4A—C5A	1.380 (3)
C21B—N1	1.477 (13)	C5A—H5A	0.9500
C22B—H22C	0.9900	C5A—C6A	1.389 (2)
C22B—H22D	0.9900	C6A—H6A	0.9500
C22B—O3B	1.463 (16)	C7A—C8A	1.394 (2)
O3B—H3B	0.84 (2)	C7A—C12A	1.406 (3)
O4—H4	0.890 (17)	C7A—B1	1.640 (3)
O4—C25	1.445 (3)	C8A—H8A	0.9500
N1—C1	1.474 (2)	C8A—C9A	1.389 (3)
N1—C11	1.470 (2)	C9A—H9A	0.9500
N2—C2	1.322 (2)	C9A—C10A	1.380 (3)
N2—C10	1.380 (2)	C10A—H10A	0.9500
N3—C12	1.325 (2)	C10A—C11A	1.375 (3)
N3—C20	1.377 (2)	C11A—H11C	0.9500
C1—H1A	0.9900	C11A—C12A	1.383 (3)
C1—H1B	0.9900	C12A—H12A	0.9500
C1—C2	1.502 (2)	C13A—C14A	1.404 (2)
C2—C3	1.408 (3)	C13A—C18A	1.394 (3)
C3—H3A	0.9500	C13A—B1	1.648 (2)
C3—C4	1.357 (3)	C14A—H14A	0.9500
C4—H4A	0.9500	C14A—C15A	1.389 (3)
C4—C5	1.410 (3)	C15A—H15A	0.9500
C5—C6	1.407 (3)	C15A—C16A	1.383 (3)
C5—C10	1.415 (2)	C16A—H16A	0.9500
C6—H6	0.9500	C16A—C17A	1.377 (3)
C6—C7	1.366 (3)	C17A—H17A	0.9500
C7—H7	0.9500	C17A—C18A	1.393 (3)
C7—C8	1.396 (3)	C18A—H18A	0.9500
C8—H8	0.9500	C19A—C20A	1.397 (2)
C8—C9	1.373 (3)	C19A—C24A	1.403 (2)
C9—H9	0.9500	C19A—B1	1.651 (2)
C9—C10	1.408 (2)	C20A—H20A	0.9500
C11—H11A	0.9900	C20A—C21A	1.393 (2)
C11—H11B	0.9900	C21A—H21E	0.9500
C11—C12	1.509 (2)	C21A—C22A	1.376 (3)
C12—C13	1.406 (2)	C22A—H22E	0.9500
C13—H13	0.9500	C22A—C23A	1.383 (3)
C13—C14	1.362 (3)	C23A—H23A	0.9500
C14—H14	0.9500	C23A—C24A	1.389 (2)
C14—C15	1.408 (2)	C24A—H24D	0.9500
C15—C16	1.416 (3)	O1S—H1S	0.8400
C15—C20	1.414 (2)	O1S—C1S	1.416 (3)
C16—H16	0.9500	C1S—H1SA	0.9800
C16—C17	1.364 (3)	C1S—H1SB	0.9800
C17—H17	0.9500	C1S—H1SC	0.9800
C17—C18	1.401 (3)		
			
O1—Mn1—O3	104.41 (14)	C14—C15—C20	117.95 (15)
O1—Mn1—O3B	107.0 (6)	C20—C15—C16	119.40 (16)
O1—Mn1—O4	89.75 (7)	C15—C16—H16	120.0
O1—Mn1—N2	108.03 (6)	C17—C16—C15	120.08 (18)
O1—Mn1—N3	102.93 (6)	C17—C16—H16	120.0
O3—Mn1—O4	83.98 (17)	C16—C17—H17	119.8
O3—Mn1—N1	78.14 (13)	C16—C17—C18	120.39 (17)
O3—Mn1—N2	86.19 (17)	C18—C17—H17	119.8
O3B—Mn1—O4	76.4 (7)	C17—C18—H18	119.4
O3B—Mn1—N1	75.0 (5)	C19—C18—C17	121.11 (17)
O3B—Mn1—N2	92.6 (8)	C19—C18—H18	119.4
O3B—Mn1—N3	143.7 (5)	C18—C19—H19	120.1
N1—Mn1—O4	86.88 (6)	C18—C19—C20	119.89 (17)
N1—Mn1—N2	75.63 (5)	C20—C19—H19	120.1
N1—Mn1—N3	73.81 (5)	N3—C20—C15	121.47 (15)
O3—Mn1—N3	149.83 (12)	N3—C20—C19	119.39 (15)
O1—Mn1—N1	175.54 (6)	C15—C20—C19	119.12 (15)
N2—Mn1—O4	161.38 (6)	O1—C23—C24	117.54 (18)
N3—Mn1—O4	83.64 (6)	O2—C23—O1	123.34 (17)
N3—Mn1—N2	97.28 (5)	O2—C23—C24	119.07 (18)
C23—O1—Mn1	137.39 (13)	C23—C24—H24A	109.5
H21A—C21—H21B	108.1	C23—C24—H24B	109.5
C22—C21—H21A	109.5	C23—C24—H24C	109.5
C22—C21—H21B	109.5	H24A—C24—H24B	109.5
N1—C21—H21A	109.5	H24A—C24—H24C	109.5
N1—C21—H21B	109.5	H24B—C24—H24C	109.5
N1—C21—C22	110.6 (3)	O4—C25—H25A	109.5
C21—C22—H22A	109.9	O4—C25—H25B	109.5
C21—C22—H22B	109.9	O4—C25—H25C	109.5
H22A—C22—H22B	108.3	H25A—C25—H25B	109.5
O3—C22—C21	109.0 (3)	H25A—C25—H25C	109.5
O3—C22—H22A	109.9	H25B—C25—H25C	109.5
O3—C22—H22B	109.9	C2A—C1A—C6A	114.92 (16)
Mn1—O3—H3	131 (3)	C2A—C1A—B1	124.23 (15)
C22—O3—Mn1	113.0 (4)	C6A—C1A—B1	120.85 (14)
C22—O3—H3	114 (3)	C1A—C2A—H2A	118.8
H21C—C21B—H21D	108.7	C3A—C2A—C1A	122.42 (18)
C22B—C21B—H21C	110.6	C3A—C2A—H2A	118.8
C22B—C21B—H21D	110.6	C2A—C3A—H3AA	119.7
N1—C21B—H21C	110.6	C4A—C3A—C2A	120.60 (18)
N1—C21B—H21D	110.6	C4A—C3A—H3AA	119.7
N1—C21B—C22B	105.9 (9)	C3A—C4A—H4AA	120.6
C21B—C22B—H22C	110.2	C5A—C4A—C3A	118.86 (17)
C21B—C22B—H22D	110.2	C5A—C4A—H4AA	120.6
H22C—C22B—H22D	108.5	C4A—C5A—H5A	120.0
O3B—C22B—C21B	107.5 (15)	C4A—C5A—C6A	120.01 (17)
O3B—C22B—H22C	110.2	C6A—C5A—H5A	120.0
O3B—C22B—H22D	110.2	C1A—C6A—H6A	118.4
Mn1—O3B—H3B	141 (10)	C5A—C6A—C1A	123.15 (16)
C22B—O3B—Mn1	112.2 (16)	C5A—C6A—H6A	118.4
C22B—O3B—H3B	90 (10)	C8A—C7A—C12A	114.20 (16)
Mn1—O4—H4	109 (2)	C8A—C7A—B1	124.47 (16)
C25—O4—Mn1	137.22 (16)	C12A—C7A—B1	121.23 (15)
C25—O4—H4	111 (2)	C7A—C8A—H8A	118.6
C21—N1—Mn1	105.73 (17)	C9A—C8A—C7A	122.81 (19)
C21B—N1—Mn1	111.4 (6)	C9A—C8A—H8A	118.6
C1—N1—Mn1	109.55 (11)	C8A—C9A—H9A	119.5
C1—N1—C21	116.0 (2)	C10A—C9A—C8A	120.9 (2)
C1—N1—C21B	98.8 (5)	C10A—C9A—H9A	119.5
C11—N1—Mn1	106.33 (10)	C9A—C10A—H10A	120.9
C11—N1—C21	109.97 (19)	C11A—C10A—C9A	118.26 (19)
C11—N1—C21B	121.5 (7)	C11A—C10A—H10A	120.9
C11—N1—C1	108.77 (14)	C10A—C11A—H11C	119.9
C2—N2—Mn1	114.17 (11)	C10A—C11A—C12A	120.2 (2)
C2—N2—C10	117.78 (15)	C12A—C11A—H11C	119.9
C10—N2—Mn1	127.93 (11)	C7A—C12A—H12A	118.2
C12—N3—Mn1	113.60 (11)	C11A—C12A—C7A	123.60 (18)
C12—N3—C20	118.50 (14)	C11A—C12A—H12A	118.2
C20—N3—Mn1	127.48 (11)	C14A—C13A—B1	121.98 (15)
N1—C1—H1A	108.5	C18A—C13A—C14A	114.98 (16)
N1—C1—H1B	108.5	C18A—C13A—B1	122.95 (15)
N1—C1—C2	115.08 (15)	C13A—C14A—H14A	118.6
H1A—C1—H1B	107.5	C15A—C14A—C13A	122.8 (2)
C2—C1—H1A	108.5	C15A—C14A—H14A	118.6
C2—C1—H1B	108.5	C14A—C15A—H15A	119.9
N2—C2—C1	118.68 (16)	C16A—C15A—C14A	120.1 (2)
N2—C2—C3	123.55 (16)	C16A—C15A—H15A	119.9
C3—C2—C1	117.66 (15)	C15A—C16A—H16A	120.5
C2—C3—H3A	120.3	C17A—C16A—C15A	119.03 (18)
C4—C3—C2	119.41 (17)	C17A—C16A—H16A	120.5
C4—C3—H3A	120.3	C16A—C17A—H17A	120.0
C3—C4—H4A	120.3	C16A—C17A—C18A	120.0 (2)
C3—C4—C5	119.39 (17)	C18A—C17A—H17A	120.0
C5—C4—H4A	120.3	C13A—C18A—H18A	118.5
C4—C5—C10	118.09 (16)	C17A—C18A—C13A	123.07 (19)
C6—C5—C4	122.50 (17)	C17A—C18A—H18A	118.5
C6—C5—C10	119.40 (16)	C20A—C19A—C24A	115.08 (15)
C5—C6—H6	119.6	C20A—C19A—B1	123.20 (15)
C7—C6—C5	120.80 (19)	C24A—C19A—B1	121.72 (14)
C7—C6—H6	119.6	C19A—C20A—H20A	118.7
C6—C7—H7	120.2	C21A—C20A—C19A	122.51 (17)
C6—C7—C8	119.66 (18)	C21A—C20A—H20A	118.7
C8—C7—H7	120.2	C20A—C21A—H21E	119.7
C7—C8—H8	119.3	C22A—C21A—C20A	120.55 (17)
C9—C8—C7	121.33 (18)	C22A—C21A—H21E	119.7
C9—C8—H8	119.3	C21A—C22A—H22E	120.6
C8—C9—H9	120.1	C21A—C22A—C23A	118.90 (17)
C8—C9—C10	119.90 (18)	C23A—C22A—H22E	120.6
C10—C9—H9	120.1	C22A—C23A—H23A	120.0
N2—C10—C5	121.73 (15)	C22A—C23A—C24A	119.99 (18)
N2—C10—C9	119.36 (16)	C24A—C23A—H23A	120.0
C9—C10—C5	118.90 (16)	C19A—C24A—H24D	118.5
N1—C11—H11A	109.4	C23A—C24A—C19A	122.97 (17)
N1—C11—H11B	109.4	C23A—C24A—H24D	118.5
N1—C11—C12	111.03 (14)	C1A—B1—C13A	108.68 (13)
H11A—C11—H11B	108.0	C1A—B1—C19A	108.83 (13)
C12—C11—H11A	109.4	C7A—B1—C1A	111.22 (13)
C12—C11—H11B	109.4	C7A—B1—C13A	110.01 (14)
N3—C12—C11	118.03 (15)	C7A—B1—C19A	108.38 (12)
N3—C12—C13	123.03 (16)	C13A—B1—C19A	109.71 (13)
C13—C12—C11	118.92 (15)	C1S—O1S—H1S	109.5
C12—C13—H13	120.4	O1S—C1S—H1SA	109.5
C14—C13—C12	119.19 (16)	O1S—C1S—H1SB	109.5
C14—C13—H13	120.4	O1S—C1S—H1SC	109.5
C13—C14—H14	120.1	H1SA—C1S—H1SB	109.5
C13—C14—C15	119.83 (16)	H1SA—C1S—H1SC	109.5
C15—C14—H14	120.1	H1SB—C1S—H1SC	109.5
C14—C15—C16	122.64 (17)		
			
Mn1—O1—C23—O2	−42.8 (3)	C16—C15—C20—C19	0.6 (2)
Mn1—O1—C23—C24	139.8 (2)	C16—C17—C18—C19	0.5 (3)
Mn1—N1—C1—C2	−29.29 (19)	C17—C18—C19—C20	−0.7 (3)
Mn1—N1—C11—C12	−43.03 (16)	C18—C19—C20—N3	−178.44 (16)
Mn1—N2—C2—C1	−6.0 (2)	C18—C19—C20—C15	0.1 (3)
Mn1—N2—C2—C3	177.88 (14)	C20—N3—C12—C11	178.90 (14)
Mn1—N2—C10—C5	−177.28 (11)	C20—N3—C12—C13	0.7 (2)
Mn1—N2—C10—C9	2.2 (2)	C20—C15—C16—C17	−0.9 (3)
Mn1—N3—C12—C11	5.76 (19)	C1A—C2A—C3A—C4A	−1.0 (3)
Mn1—N3—C12—C13	−172.45 (13)	C2A—C1A—C6A—C5A	1.9 (3)
Mn1—N3—C20—C15	170.56 (12)	C2A—C1A—B1—C7A	−110.51 (19)
Mn1—N3—C20—C19	−10.9 (2)	C2A—C1A—B1—C13A	10.7 (2)
C21—C22—O3—Mn1	37.8 (4)	C2A—C1A—B1—C19A	130.17 (18)
C21—N1—C1—C2	90.3 (2)	C2A—C3A—C4A—C5A	0.9 (3)
C21—N1—C11—C12	−157.0 (2)	C3A—C4A—C5A—C6A	0.5 (3)
C22—C21—N1—Mn1	43.1 (3)	C4A—C5A—C6A—C1A	−2.0 (3)
C22—C21—N1—C1	−78.6 (3)	C6A—C1A—C2A—C3A	−0.4 (3)
C22—C21—N1—C11	157.5 (2)	C6A—C1A—B1—C7A	69.80 (19)
C21B—C22B—O3B—Mn1	−52.1 (17)	C6A—C1A—B1—C13A	−168.96 (15)
C21B—N1—C1—C2	87.2 (7)	C6A—C1A—B1—C19A	−49.5 (2)
C21B—N1—C11—C12	−171.7 (6)	C7A—C8A—C9A—C10A	0.2 (4)
C22B—C21B—N1—Mn1	−36.3 (13)	C8A—C7A—C12A—C11A	−0.4 (3)
C22B—C21B—N1—C1	−151.4 (10)	C8A—C7A—B1—C1A	−23.7 (2)
C22B—C21B—N1—C11	90.1 (11)	C8A—C7A—B1—C13A	−144.17 (16)
N1—C21—C22—O3	−55.1 (5)	C8A—C7A—B1—C19A	95.89 (19)
N1—C21B—C22B—O3B	56.6 (17)	C8A—C9A—C10A—C11A	0.1 (4)
N1—C1—C2—N2	24.6 (2)	C9A—C10A—C11A—C12A	−0.5 (3)
N1—C1—C2—C3	−159.00 (16)	C10A—C11A—C12A—C7A	0.7 (3)
N1—C11—C12—N3	26.3 (2)	C12A—C7A—C8A—C9A	0.0 (3)
N1—C11—C12—C13	−155.37 (16)	C12A—C7A—B1—C1A	160.32 (15)
N2—C2—C3—C4	0.2 (3)	C12A—C7A—B1—C13A	39.9 (2)
N3—C12—C13—C14	1.0 (3)	C12A—C7A—B1—C19A	−80.09 (18)
C1—N1—C11—C12	74.87 (17)	C13A—C14A—C15A—C16A	0.8 (3)
C1—C2—C3—C4	−176.00 (18)	C14A—C13A—C18A—C17A	−0.9 (3)
C2—N2—C10—C5	−1.5 (2)	C14A—C13A—B1—C1A	−85.78 (19)
C2—N2—C10—C9	177.96 (16)	C14A—C13A—B1—C7A	36.2 (2)
C2—C3—C4—C5	−1.9 (3)	C14A—C13A—B1—C19A	155.34 (15)
C3—C4—C5—C6	−178.02 (18)	C14A—C15A—C16A—C17A	−1.0 (3)
C3—C4—C5—C10	1.9 (3)	C15A—C16A—C17A—C18A	0.2 (3)
C4—C5—C6—C7	179.84 (18)	C16A—C17A—C18A—C13A	0.7 (3)
C4—C5—C10—N2	−0.2 (2)	C18A—C13A—C14A—C15A	0.1 (3)
C4—C5—C10—C9	−179.62 (16)	C18A—C13A—B1—C1A	90.51 (18)
C5—C6—C7—C8	−0.5 (3)	C18A—C13A—B1—C7A	−147.51 (16)
C6—C5—C10—N2	179.71 (15)	C18A—C13A—B1—C19A	−28.4 (2)
C6—C5—C10—C9	0.3 (2)	C19A—C20A—C21A—C22A	0.4 (3)
C6—C7—C8—C9	0.8 (3)	C20A—C19A—C24A—C23A	0.4 (2)
C7—C8—C9—C10	−0.6 (3)	C20A—C19A—B1—C1A	148.13 (15)
C8—C9—C10—N2	−179.42 (17)	C20A—C19A—B1—C7A	27.0 (2)
C8—C9—C10—C5	0.0 (3)	C20A—C19A—B1—C13A	−93.08 (18)
C10—N2—C2—C1	177.65 (15)	C20A—C21A—C22A—C23A	−0.1 (3)
C10—N2—C2—C3	1.5 (2)	C21A—C22A—C23A—C24A	0.0 (3)
C10—C5—C6—C7	0.0 (3)	C22A—C23A—C24A—C19A	−0.2 (3)
C11—N1—C1—C2	−145.12 (15)	C24A—C19A—C20A—C21A	−0.5 (2)
C11—C12—C13—C14	−177.15 (16)	C24A—C19A—B1—C1A	−32.7 (2)
C12—N3—C20—C15	−1.5 (2)	C24A—C19A—B1—C7A	−153.78 (15)
C12—N3—C20—C19	177.03 (15)	C24A—C19A—B1—C13A	86.09 (18)
C12—C13—C14—C15	−1.9 (3)	B1—C1A—C2A—C3A	179.91 (19)
C13—C14—C15—C16	−177.40 (17)	B1—C1A—C6A—C5A	−178.38 (16)
C13—C14—C15—C20	1.1 (3)	B1—C7A—C8A—C9A	−176.23 (19)
C14—C15—C16—C17	177.64 (18)	B1—C7A—C12A—C11A	175.92 (16)
C14—C15—C20—N3	0.6 (2)	B1—C13A—C14A—C15A	176.65 (17)
C14—C15—C20—C19	−177.92 (16)	B1—C13A—C18A—C17A	−177.39 (17)
C15—C16—C17—C18	0.3 (3)	B1—C19A—C20A—C21A	178.73 (16)
C16—C15—C20—N3	179.20 (16)	B1—C19A—C24A—C23A	−178.84 (16)

### (Acetato-κO)[2-hydroxy-N,N-bis(quinolin-2-ylmethyl)ethanamine-κ4N,N',N'',O](methanol-κO)manganese(II) tetraphenylborate methanol monosolvate (2) Hydrogen-bond geometry (Å, º)

**Table:**

*D*—H···*A*	*D*—H	H···*A*	*D*···*A*	*D*—H···*A*
O3—H3···O2^i^	0.85 (2)	1.79 (2)	2.631 (8)	170 (4)
O3*B*—H3*B*···O2^i^	0.84 (2)	1.87 (8)	2.65 (3)	152 (14)
O4—H4···O1*S*	0.89 (2)	1.77 (2)	2.646 (2)	168 (3)
C9—H9···O1	0.95	2.43	3.325 (3)	157
C17—H17···O1*S*^ii^	0.95	2.73	3.364 (3)	125
C18—H18···O1*S*^ii^	0.95	2.73	3.367 (2)	125
C19—H19···O1	0.95	2.39	3.183 (2)	141
C25—H25*A*···N3	0.98	2.79	3.387 (3)	120
O1*S*—H1*S*···O2^i^	0.84	1.92	2.691 (2)	151

Symmetry codes: (i) −*x*, −*y*+1, −*z*+1; (ii) *x*+1, *y*, *z*.

### Selected bond lengths (Å) and angles (°) of [1](BPh4)2·(CH2Cl2)1.45

**Table:**

Mn1–O1	2.3255 (12)
Mn1–O2	2.0617 (13)
Mn1–O3A	2.0908 (14)
Mn1–N1	2.3179 (14)
Mn1–N2	2.2730 (14)
Mn1–N3	2.3588 (16)
N2–Mn1–N3	73.25 (5)
N2–Mn1–N1	75.56 (5)
N1–Mn1–N3	148.35 (5)
N2–Mn1–O1	75.32 (5)
O2–Mn1–N2	157.89 (6)
O3A–Mn1–O1	163.58 (6)

### Selected bond lengths (Å) and angles (°) of [2]BPh4·CH3OH

**Table:**

Mn1—O1	2.0551 (14)
Mn1—O3	2.182 (7)
Mn1—O3B	2.13 (3)
Mn1—O4	2.3190 (16)
Mn1—N1	2.2787 (15)
Mn1—N2	2.3167 (15)
Mn1—N3	2.2664 (14)
N1—Mn1—N2	75.63 (5)
N1—Mn1—N3	73.81 (5)
O3—Mn1—N3	149.83 (12)
O1—Mn1—N1	175.54 (6)
N2—Mn1—O4	161.38 (6)
